# Current Approaches and Challenges in Advanced Oxidation Processes for Nanoplastic Degradation

**DOI:** 10.1002/advs.202504352

**Published:** 2025-06-25

**Authors:** Arezou Fazli, Athanassia Athanassiou, Despina Fragouli

**Affiliations:** ^1^ Smart Materials Istituto Italiano di Tecnologia via Morego 30 Genova 16163 Italy; ^2^ Institut de Chimie et Procédés pour l’Energie, l’Environnement et la Santé (ICPEES) CNRS/University of Strasbourg 25 rue Becquerel Strasbourg 67087 France

**Keywords:** environmental fate, ozonation, photocatalysis, plastics, pollution, remediation

## Abstract

The proliferation of plastic production has led to a surge in nanoplastic (NPs) pollution, posing significant environmental and health risks. Despite efforts to mitigate plastic waste, NPs persist as a significant challenge due to their small size, high surface‐to‐volume ratio, and complex nature. This review analyzes existing research on advanced oxidation processes, focused exclusively on NPs remediation, including ozonation, electrochemical, photocatalytic, and plasma‐induced processes. Gaps in the development of effective processes and analytical methods for the treatment of such plastic particles with sizes lower than 1 µm are highlighted, and future directions are suggested. This study aims to improve understanding and support sustainable solutions for the remediation of NPs from contaminated water sources, in alingment with the United Nations' Sustainable Development Goal 6.

## Graphical abstract

This review explores recent progress in advanced oxidation processes (AOPs) for the degradation of nanoplastics (NPs) in water. It presents a comparative assessment of key AOP technologies and identifies current limitations in analytical methods and treatment efficiency. The work highlights critical challenges and proposes future directions to support sustainable water treatment solutions.

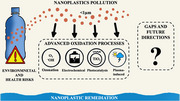

## 1 Introduction

The introduction of the concept of polymerization by Herman Staudinger in 1920 led to a better understanding of polymer chemistry and, therefore, to the more massive production of plastic worldwide^[^ ^1^ ^]^ Plastic has multifaceted properties, such as low thermal conductivity, lightweight, ease of preparation, ability to take different forms and shapes, and tunable mechanical properties.^[^ ^2^ ^]^ For these reasons, plastic is an essential component of various materials in applications such as agriculture, textiles, medical and electronic devices, personal care products, vehicles, packaging, toys, etc.^[^ ^3^ ^]^ The high demand for plastic materials is reflected by the scale of global plastic production, which has significantly increased over the past 60 years, from 15 million tons in 1964 to 400.3 million tons in 2022. Should this trend persist, production is projected to double within the next years .^[^ ^4^, ^5^, ^6^ ^]^ On top of this, recent research studies indicate that the COVID‐19 pandemic increased even more plastic needs.^[^ ^7^ ^]^

Although the plastic production continues to increase, the recycling rate remains low. Studies have shown that only 9% of the yearly plastic waste is recycled, with the rest ending up in the environment through various pathways.^[^ ^8^, ^9^ ^]^ Even though multiple efforts have been made so far to pose some management policies for the limitation of plastic waste through the control of plastic production, utilization and end of life, the use of biodegradable plastics, and the improvement of the recycling processes,^[^ ^3^ ^]^ there is still a considerable amount of plastic waste present in the environment.

Oceans and rivers are estimated to receive nearly 13 million tons of plastic waste annually.^[^ ^10^ ^]^ Plastic debris in water bodies is present in the form of macroplastics (> 1cm), mesoplastics (1–10 mm), microplastics (MPs, 1–1000 µm), and nanoplastics (NPs). Plastic particles with sizes below 1 µm are generally considered as NPs; however, for the sake of conformity with the definitions of nanomaterials, Hartmann et al.^[^ ^11^ ^]^ recently proposed the subdivision into submicron‐plastics with sizes ranging between 100 nm and 1 µm and NPs with sizes below 100 nm. Nonetheless, the present review considers NPs as plastic particles with sizes smaller than 1 µm.

Recent research suggests that MPs and NPs exacerbate environmental pollution due to their destructive effects on aquatic organisms and human health. It has been proven that MPs and NPs can affect the growth rate of marine organisms and enhance their mortality rate, while on a cellular scale, they can cause gene expression variations and a high cell death rate.^[^ ^12^, ^13^ ^]^ NPs and MPs may accumulate in the organs and cause some disorders in living bodies. Furthermore, due to their small size, NPs can quickly enter the bloodstream, permeate cellular barriers or cell membranes, and alter their functionalities.^[^ ^12^ ^]^

For the above reasons, NPs are emerging as water pollutants of significant concern, even in low environmental concentrations. Due to their characteristics, performing on‐site detection and monitoring, studying their environmental and biological fate, and evaluating processes for their remediation are challenging tasks. Various advanced characterization methods have been developed to contrast the difficulties of detecting and analyzing such organic particles.^[^ ^14^ ^]^ These methodsdemonstrate their capability to efficiently track small amounts of NPs in impure samples.^[^ ^14^, ^15^ ^]^ Although significant steps forward have been made, current studies on the NPs’ fate are mainly conducted using artificial NPs and controlled environments, with the risk of missing some critical parameters for the transition from the laboratory to environmental conditions.

Due to their small size, complicated surface chemistry, non‐biodegradable nature, and presence in complex water bodies, environmentally relevant NPs are very difficult to remove from the water environment. Nonetheless, recent studies have demonstrated the ability to remediate NPs of well‐known chemistry in controlled, artificial water environments through adsorption/aggregation,^[^ ^16^, ^17^ ^]^ coagulation,^[^ ^18^ ^]^ flocculation,^[^ ^19^, ^20^ ^]^ sedimentation,^[^ ^21^ ^]^ biological^[^ ^22^ ^]^ treatments and membrane filtration.^[^ ^23^ ^]^ Although the performance of these methods is promising, in most cases, NPs are only partially removed from water samples. At the same time, some of the remediation processes, such as coagulation/flocculation, require the presence of additional chemicals in the systems, which may produce secondary pollution. To address these problems, advanced oxidation processes (AOPs) have been recently reported to be among the most promising methods for the remediation of NPs, as they can provoke the mineralization of the plastic particles while producing limited secondary pollution.^[^ ^24^ ^]^

AOPs, such as electrochemical,^[^ ^25^ ^]^ ozonation,^[^ ^26^ ^]^ and photocatalytic^[^ ^27^, ^28^, ^29^, ^30^ ^]^ processes, are newly emerged methods in the field of NPs degradation. However, the small size of the NPs and their complex organic nature limit their characterization and analysis during the treatment process. Therefore, diagnosing potential problems to achieve highly efficient degradation of NPs using AOPs remains a challenge. To do so, data collected from recent studies should be reported, compared, and critically commented on.

While previous review papers have effectively examined the role of AOPs in environmental remediation,^[^ ^15^, ^31^, ^32^, ^33^ ^]^ they have primarily focused on MPs, with limited discussion on their efficiency in degrading nano‐sized plastic particles (<1 µm). Moreover, existing reviews often provide broad overviews but lack a comparative analysis of different AOPs regarding degradation efficiency, challenges, and real‐world applicability. This review uniquely focuses on the application of AOPs for NPs degradation, critically evaluates recent experimental findings, highlights the latest advancements in NPs characterization techniques, and presents a forward‐looking perspective on future research directions to enhance the feasibility of these methods for real‐world water treatment applications. This paper aims to provide a roadmap for future investigations into NPs remediation using AOPs by synthesizing recent studies and identifying research gaps. In this context, we first focus on I) the sources of NPs and their hazards as water contaminants, II) the use of different remediation strategies for the degradation of NPs, III) the applied AOPs on the in situ degradation of NPs, and IV) the current research gaps and perspectives in degrading NPs through AOPs. With this review paper, we aim to highlight and optimize the potential of sustainable AOP methods in the degradation of NPs and to give an overall view of the effective characterization and analysis methods to promote accuracy in this field.

## 2 Principal Sources and Hazards of NPs

MPs and NPs are classified as primary or secondary depending on their sources. Particles deriving from primary sources are components of commercial products, and they enter the environment directly through these products. Industrial abrasives, cosmetics, and exfoliating personal care products, adhesives, electronics, paints, and drug delivery medical devices are well‐known examples of commercially available products that contain primary MPs and NPs.^[^ ^12^, ^13^, ^34^ ^]^ Alternatively, all the discarded plastic products, such as plastic bags, bottles, ropes, fishing nets, food packaging, and electronics, can be sources of secondary MPs and NPs.^[^ ^34^, ^35^ ^]^ These particles derive from the breakdown of plastic debris caused by physical, chemical, and biological actions such as hydrolysis, photodegradation, mechanical abrasion, and biodegradation.^[^ ^35^, ^36^ ^]^ For instance, Lambort et al.^[^ ^37^ ^]^ proved that polystyrene (PS) coffee cups could be degraded to MPs and subsequently to NPs under ultraviolet (UV) and visible light irradiation. In addition, Prenner et al.^[^ ^38^ ^]^ demonstrated that rubber tires can produce NPs during constant friction, particularly in driving conditions (high speed and sharp braking).^[^ ^39^ ^]^ Accordingly, a study has shown that in 2018 in Austria, ≈6% of the used rubber tires were emitted into the air as micro‐ and nano‐sized particles.^[^ ^38^ ^]^ Moreover, Dawson et al.^[^ ^40^ ^]^ reported that the Antarctic krill can transform MPs into NPs through digestive fragmentation, proving that NPs can be generated by ingesting MPs in marine species.

**Figure 1:**
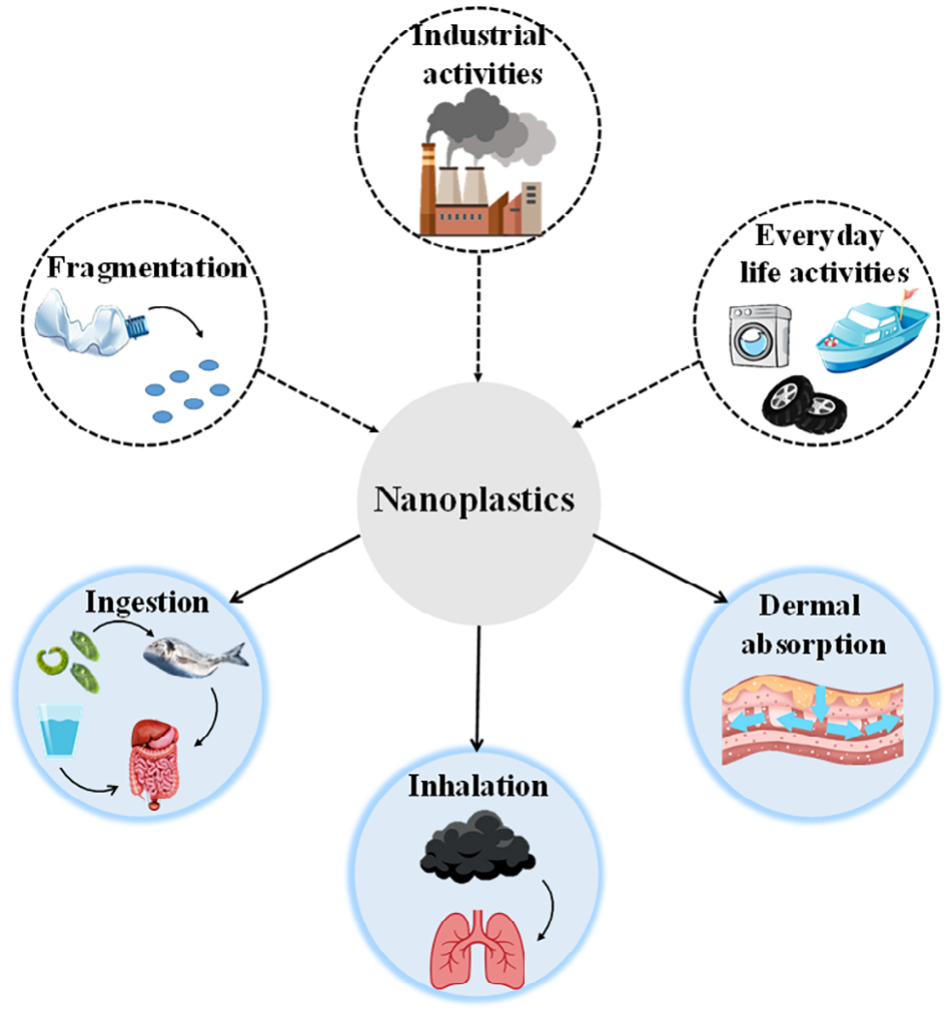
A general summary of sources producing NPs in water and pathways to human exposure.

Regarding environmental pollution, the presence of NPs in water has become an emerging environmental issue. **Figure** **1** provides an overview of the significant sources of NPs in water. Water bodies are polluted by macroplastics and MPs, which originate from both sea‐based (e.g., fishing gears) and land‐based activities, such as inadequate waste disposal and management, industrial operations, municipal sewage and sludge, tire abrasion, and surface waters runoff, eventually passing to the oceans through various pathways including wind, water, and soil^[^ ^41^, ^42^ ^]^ Chemical, physical, and biological transformation of such plastic litter in water results in NPs.^[^ ^36^, ^40^ ^]^ In addition, although wastewater treatment plants (WWTPs) limit the dispersion of water pollutants, they can be a significant source of MPs and NPs. Concerning MPs, it is estimated that 25% of the globally released MPs in the environment are attributed to WWTPs.^[^ ^10^ ^]^ In the case of NPs, although there is no precise information about the amount, such small particles can undoubtedly pass through the various water treatment stages and be released together with the MPs in the environment, or can even be generated from the fragmentation of the MPs through shear stress forces during the treatment processes.^[^ ^10^ ^]^

A recent study on the biological fate of PS NPs of two different sizes (i.e., 50 and 500 nm) showed that the NPs of larger size (500 nm) can be accumulated in the digestive organs of aquatic animals. In contrast, smaller NPs (50 nm) can penetrate tissues, cause immune system disorders, and affect marine organisms' growth rates.^[^ ^43^ ^]^ In addition, the functional groups and the high surface area of NPs allow the adsorption and transport of various water pollutants such as heavy metals or endocrine disruptors as well as the effective release of various endogenous pollutants originating from the components and additives used in the production of plastic.^[^ ^44^ ^]^

Concerning human exposure, as depicted in Figure 1, NPs may enter the human organs through ingestion, inhalation, and dermal absorption.^[^ ^45^ ^]^ Dermal absorption of NPs can occur using personal skin care products containing such NPs. On the other hand, ingestion of NPs is likely the main pathway of entry into human organs since they can be ingested by consuming contaminated food, like seafood, salt, and beer, or by drinking contaminated water.^[^ ^45^, ^46^ ^]^ Their small size, high surface area, and surface charge raise diverse adverse effects. Recent experimental findings suggest that NPs can contribute to the development of neurodegenerative diseases. In vivo studies on mice proved that the oral ingestion of NPs (30–50 nm) may reach the brain tissue and induce cognitive dysfunction.^[^ ^47^ ^]^ Moreover, in vitro studies demonstrated that NPs (30 nm) can penetrate human neural stem cells through endocytosis, where they accumulate, leading to decreased cell proliferation and eventually death.^[^ ^48^ ^]^ Marfella et al.^[^ ^49^ ^]^ carried out an in‐depth observational study on the correlation between the presence of MPs and NPs in the carotid artery and a higher risk of heart attack, stroke, or death. To do so, the attention has been focused on patients undergoing surgery for carotid artery disease. Upon analysis, it was found that out of 257 patients, 150 had polyethylene particles in their carotid artery plaques (21.7 ± 24.5 µg mg^−1^ of plaque), while 31 patients had polyvinyl chloride (5.2 ± 2.4 µg mg^−1^ of plaque). These particles were reported to be less than 1 µm in size, likely within the nanometer range.

## 3 Remediation Strategies for NPs Contamination in Water

There is increasing evidence that drinking water is contaminated with plastic particles, making it a significant source of human exposure.^[^ ^50^ ^]^ Kosuth et al.^[^ ^51^ ^]^ studied the presence of MPs in tap and bottled water. Samples from 14 countries were subjected to vacuum filtration (cut‐off: 2.5 µm), and the filters were visually assessed using a dissecting microscope. The results showed that 81% and 93% of the tap and bottled water samples, respectively, were contaminated with MPs, mostly with fibers of length ranging from 0.1 to 5.0 mm. Moreover, Li et al.^[^ ^50^ ^]^ have recently shown the existence of NPs in tap water. The collected samples, subjected to successive filtration steps, revealed the presence of NPs with concentrations ranging between 1.67 and 2.08 µg L^−1^, depending on their size (from 255 to 58 nm), with most of them composed of PS, polyvinyl chloride, polyolefins, and polyamide. Furthermore, Huang et al.^[^ ^52^ ^]^ analyzed polyethylene terephthalate (PET) bottled water using a tangential flow ultrafiltration system. The final retentate fluid was examined through a new nanorod‐M super‐resolution optical microscopy system equipped with a super‐resolution microsphere amplifying lens. The analyses revealed the presence of organic particles in the water with sizes ranging from the micron scale to the nanoscale. In brief, NPs can be found in potable water, either surviving different water treatment processes or being released from the plastic bottles in which they are stored. Therefore, there is an essential need to control and eliminate their presence through effectivewater treatment processes. This section reviews some recently published papers on removing NPs following standard water treatment methods like coagulation, flocculation, sedimentation, filtration, adsorption, and bioremediation.

Since coagulation or flocculation methods are the typical first steps in water purification, they have also been considered for removing NPs from contaminated water. Chen et al.^[^ ^20^ ^]^ declared the high potential of Mg and Al‐based layered double hydroxides (Mg/Al‐LDHs) for the flocculation of PS NPs with sizes ranging between 50 and 100 nm. In particular, it has been demonstrated that during the flocculation process, at specific pH values, Mg/Al‐LDHs are formed in situ and, simultaneously, during their formation, can capture existing PS NPs in their layers. Such Mg/Al flocculants can remove ≈90.0% of the PS NPs from the liquid media. However, a significant challenge for the practical application of this method is the need to separate the LDH‐encapsulated NPs from the treated water. Alternatively, Zhang et al.^[^ ^18^ ^]^ studied the removal of fluorescent PS NPs (180 nm) from drinking water through coagulation‐flocculation combined with sedimentation (CFS). Fluorescent PS allowed for easier monitoring of the NPs and facilitated tracking of their environmental fate, providing valuable insights into their behavior during the treatment process. As reported, even with the aid of coagulants, NPs were not removed upon the application of CFS. Their results suggest a general rule that such water treatment processes are inefficient at removing hydrophobic NPs which easily float on water due to their low density. Nonetheless, the same study proved the high ability of column filtration to remove NPs (180 nm) with an efficiency of 99%, as due to their small size, they are likely to attach to (or be absorbed by) the grains. Although this method works well, some NPs still pass through the filter and can be found in water. Particle size significantly influenced removal efficiency, while the relationship was not strictly linear. The lowest efficiency (87%) was observed for intermediate‐sized particles (10–20 µm), whereas both smaller (180 nm) and larger (106–125 µm) particles showed higher removal rates (99% and 100%, respectively). On top of this, the used NPs were composed of pure polymer and had a homogeneous spherical shape. In environmental conditions, the NPs may arrive at much smaller sizes, while they are most likely characterized by shape inhomogeneity and altered surface chemistry. As previously referred, plastic fragments in water are exposed to different degradation processes and interactions with various components found in the environment, resulting in increased hydrophilicity or the formation of complexes. For instance, unlike pristine NPs, those in the environment undergo natural aging processes such as UV irradiation or biological degradation. These processes change the surface chemistry, introducing oxygen‐containing functional groups.^[^ ^53^ ^]^ Furthermore, in the case of environmental samples, NPs and other organic and inorganic components may clog the filters and decrease their efficiency for continuous water treatment.^[^ ^54^ ^]^

Alternatively, various batch adsorption studies were conducted to assess the effectiveness of removing NPs from water. Ganie et al.^[^ ^55^ ^]^ prepared sugarcane bagasse‐derived biochar powders at different pyrolysis temperatures, and explored their efficiency in adsorbing hydrophilic PS‐based Latex beads (size: a few hundred nm < 500 nm). The biochar prepared at the highest pyrolysis temperature (750 °C) had a high surface area (540.36 m^2^ g^−1^) and an enhanced pore volume. At the same time, the performed analysis confirmed that it possessed the least negatively charged functional groups, all characteristics that improve the electrostatic interaction with the negatively charged NPs and their entrapment into the porous structure, reaching a maximum adsorption capacity of 44.9 mg g^−1^ in deionized water dispersions. In another work, Tiwari et al.^[^ ^56^ ^]^ studied the efficiency of Zn/Al LDH to adsorb hydrophilic PS NPs (55 nm). They reported that the zeta potential of LDH (45.5 ± 0.6 mV) became negative after the interaction with NPs (‐36.8 ± 2 mV). At the same time, new absorption bands in the Fourier Transform Infrared Spectroscopy (FTIR) spectra appeared, and the surface area of the LDHs was enhanced, all supporting these structures' successful adsorption of NPs. Under optimized conditions, the maximum adsorption capacity reached was 164.49 mg g^−1^ in deionized water.

**Table** **1** shows the most relevant studies on removing NPs of sizes lower than 500 nm through adsorption processes. Although the results are promising, some restrictions still make this method challenging. For instance, in recent studies, the adsorbents were in the form of particles dispersed in the reaction media, making their recovery after the remediation process, as centrifuging, filtration, or other separation steps, necessary for their removal. Moreover, only a few studies investigated the fate of the adsorbed NPs after collecting the adsorbents, following chemical desorption steps, and examining their reusability. The complexity of the media in which the NPs are found is still an issue. Even if some studies have analyzed how the adsorption performance is affected by the presence of different components in the water medium, further studies in more complex environments are needed. In addition, most of the presented studies target NPs made of pure PS of various sizes and with specific surface chemistry. However, it remains unclear whether NPs of different nature and surface functional groups will exhibit the same behavior under similar conditions.

**Table 1:** Applied adsorbents for removing NPs from contaminated water.

Adsorbent	Adsorbent concentration [g L^−1^]	Type of NPs^a)^	Particle size [nm]	NPs concentration mg L^−1^	Removal capacity [mg g^−1^]	Refs.
CuNi@C	0.3	PS	100	10	41.93	[58]
Fly ash modified with Fe ions.	0.02	PS	80	20	88.5	[59]
Mesoporous biochar	0.4	PS	100	20	56.02	[60]
Sugarcane bagasse‐derived biochar (750 °C)	1.5	PS	<500	10	44.9	[55]
Zn/Al LDH	5	PS	55	250	164.49	[56]

^a)^ In all studies, researchers used commercially available polystyrene NPs.

Biological degradation is another attractive method for the remediation of plastic particles. Focused on NPs, Vogel et al.^[^ ^22^ ^]^ studied the enzymatic hydrolysis of PET NPs mimicking real‐world aquatic conditions through the thermophilic cutinase TfCut2 from *Thermobifida fusca*, demonstrating the effective cleavage of the polymer's ester bonds. However, the enzymatic and microbial degradation of NPs depends on the plastics' physicochemical characteristics and on the specific genes of the microorganisms required to break them down.^[^ ^57^ ^]^ Consequently, it is necessary to study the efficiency of biodegradation. Specific studies are needed for different types of NPs in complex environments to better evaluate the performance of enzymatic biodegradation.

The above‐mentioned methods for removing plastic particles from water are often time‐consuming and complex, necessitating significant space and large amounts of chemicals. Consequently, these factors limit their applicability.^[^ ^25^, ^61^ ^]^ Additionally, these methods cannot thoroughly eliminate severe and complicated water contaminants, such as plastic debris, allowing them to be quickly discharged from the wastewater treatment plants into the environment.

### 3.1 Advanced Oxidation Processes

**Figure 2:**
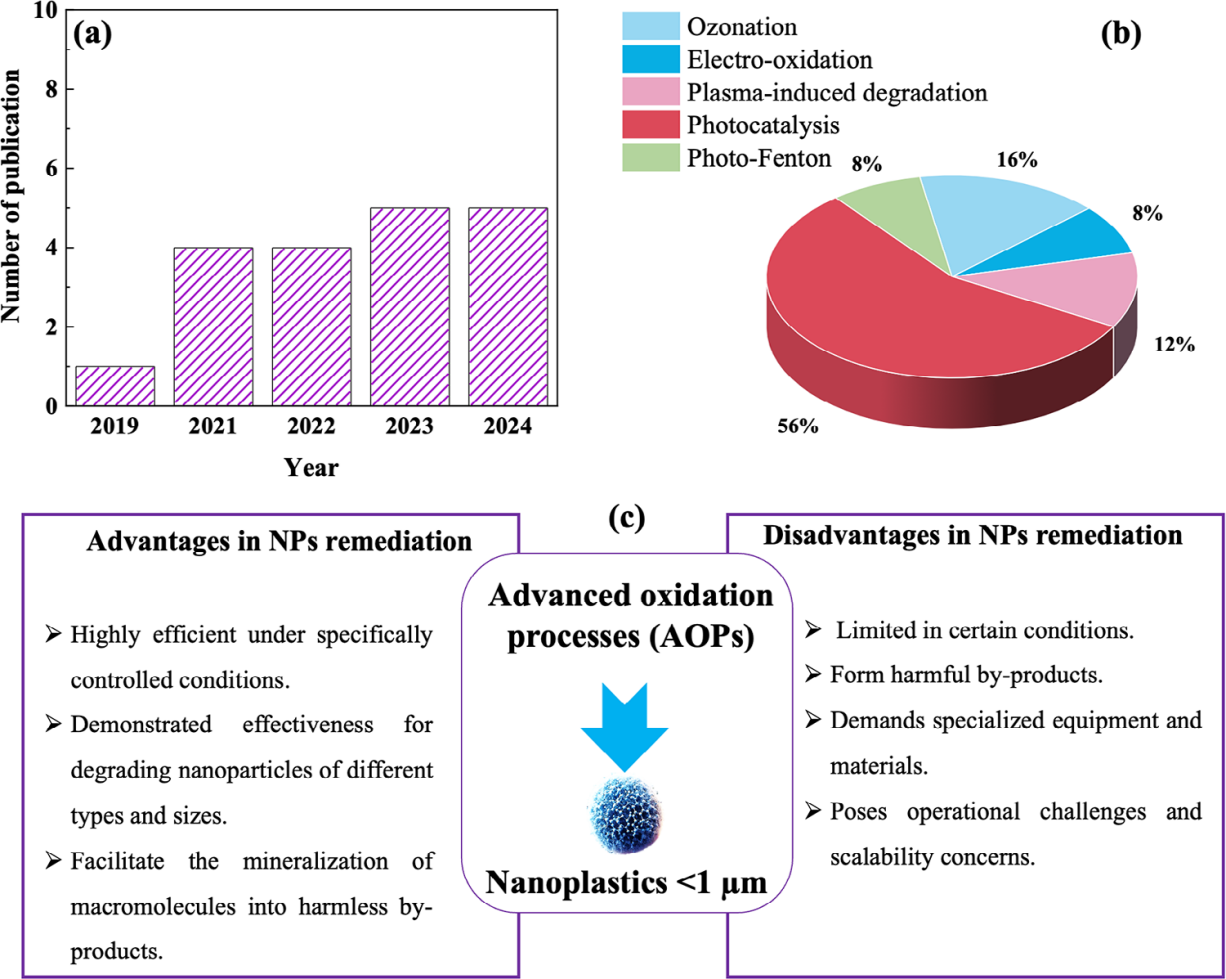
a) The annual number of research papers (excluding reviews) published between 2019 and 2024 on AOPs for NPs (<1 µm) degradation, identified through a Google Scholar search using keywords such as “advanced oxidation processes” and “degradation of nanoplastics.” b) The percentage distribution of publications based on the different types of AOPs applied. c) General advantages and disadvantages of the AOPs for NPs degradation.

AOPs have emerged as alternative methods for effectively remediating plastic particles in aqueous environments.^[^ ^62^ ^]^ These processes rely on the in situ generation of hydroxyl radicals (^•^OH), which initiate non‐selective oxidation reactions that degrade various organic pollutants with high‐rate constants. Depending on the type of AOPs, other reactive chemical species can also be generated, such as superoxide anions (O₂^•^⁻), sulfate radicals (SO₄^•⁻^), singlet oxygen (^¹^O₂), and hydroperoxyl radicals (HO₂^•^), which can contribute to the degradation of toxic compounds into harmless by‐products.^[^ ^62^, ^63^ ^]^ Focused on NPs, as shown in **Figure** **2**a, the research on the efficiency of AOPs for their degradation, although it appears to be in a preliminary stage,^[^ ^64^, ^65^, ^66^, ^67^ ^]^ has increased progressively^[^ ^26^, ^27^, ^68^, ^69^ ^]^ over the years. Notably, many of these studies focus on photocatalytic degradation, which is recognized as a sustainable and promising approach for both the degradation and mineralization of NPs (Figure 2b).

AOPs degrade NPs through distinct radical generation mechanisms, each offering unique advantages and limitations (Figure 2c). In particular, ozonation initiates degradation by decomposing ozone (O_3_) into ^•^OH and other reactive oxygen species (ROS), which drive oxidative breakdown. Similarly, electro‐oxidation facilitates both direct and indirect NPs degradation through anodic reactions that generate powerful oxidative species. Plasma‐induced degradation takes a different approach, utilizing energetic electrons, ionized gases, and UV radiation to form reactive radicals, promoting fragmentation and mineralization. In contrast, photocatalysis leverages semiconductor materials such as TiO₂ and ZnO, where light activation triggers the formation of highly reactive species that facilitate degradation. Meanwhile, the photo‐fenton process enhances radical production by combining iron catalysis with UV irradiation and hydrogen peroxide, yielding hydroxyl radicals with strong oxidative potential. Despite their varying efficiencies and operational constraints, the referred AOPs collectively offer promising pathways for addressing NPs contamination in environmental systems.

Nonetheless, significant challenges remain in elucidating the degradation mechanism and the formation of by‐products and identifying the most appropriate characterization methods for NPs subjected to AOP treatments. Moreover, many existing studies lack critical parameters and fail to adhere to standardized protocols, limiting the comparability of findings and hindering the transition from laboratory‐scale research to large‐scale applications. As summarized in **Table** **2**, by addressing these key observations and critical gaps, this work aims to provide valuable insights for future research, guiding the selection and optimization of the most promising AOPs for NPs degradation. A detailed discussion regarding the effectiveness of these methods in the remediation of NPs in water will be provided in the subsequent sections.

**Table 2:** Summary of AOPs for NPs (< 1µm) degradation: Experimental conditions, key observations, and challenges, reflecting findings from studies published between 2019 and 2024. (Please note that in the table, PS, PET, GC‐MS, Py‐GC/MS, TOC, XPS, SEM, TEM, and PMS refer to polystyrene, polyethylene terephthalate, gas chromatography‐mass spectrometry, pyrolysis‐gas chromatography/mass spectrometry, total organic carbon, X‐ray photoelectron spectroscopy, scanning electron microscopy, transmission electron microscopy, and peroxymonosulfate, respectively).

AOPs	Experimental Conditions	Removal Efficiency [%]	Key Observations and Challenges	[Ref]
Ozonation	NPs Type: Commercial PS (100 nm) [NPs]: 2.5 µg L^−1^ [O_3_]: 4.1 mg L^−1^ pH: 6.43 Reaction time: 240 min Tested in distilled water	Mineralization^b)^: 42.7%	Effective in surface modification and breakdown of polymer structure, but incomplete mineralization.	[26]
	NPs Type: Synthesized Pd‐ labeled PS (> 100) [NPs]: 1.7 mg L^−1^ [O_3_]: 5 mg L^−1^ pH: 7.90 Reaction time: 45 min Tested in lake water	No degradation was observed.	Ineffective in complex water matrices (lake water). No changes in particle morphology were observed.	[70]
Catalytic ozonation	NPs Type: PS (140 nm) [NPs]: 20 mg L⁻¹ [O_3_]: 10 mg NL⁻¹ Catalyst: Co^2^⁺ (1 mm) pH: 3 Reaction time: 120 min Tested in ultrapure water	• Mineralization^a)^: 70% Turbidity reduction: 65%	Single ozonation resulted in poor mineralization (16%) and moderate turbidity reduction (33%), highlighting the importance of the presence of the catalyst. The size of PS NPs reduced by over 99% within 5 min, raising concerns about the generation of smaller plastic fragments. Further research should focus on immobilizing transition metal catalysts for better recovery and reuse.	[71]
	NPs Type: PS (105 nm) [NPs]: 2.22 ± 0.01 mg L⁻¹ Catalyst: CeO_x_@MnO_x_ (2 mg L⁻¹) pH: 6.8–7.1 Reaction time: 50 min Tested in ultrapure water	• Molecular weight reduction: 96.70%	Ozonation alone had limited efficiency, removing only 31.67% of molecular weight in the first 10 min. ROS (^•^OH, ^•^O₂⁻, ¹O₂) played a crucial role. Repeated experiments confirmed high stability (molecular weight reduction of 95.25–96.70%). The potential toxicity of the generated by‐products should be thoroughly investigated to ensure environmental safety and sustainability.	[72]
Electro‐oxidation	NPs Type: Commercial PS (100 nm) [NPs]: 10 mg L^−1^ [Na_2_SO_4_]: 0.007 M pH: 5.00 Reaction time: 380 min Current density: 36 mA cm^−1^ Anode: Boron‐doped diamond Cathode: Titanium and carbon felt	• With titanium cathode: Mineralization: 56.2% Degradation: 80.4% • With carbon felt: Mineralization: 63.8% Degradation: 92.8%	Significant mineralization and polymer breakdown were achieved, with the carbon felt cathode outperforming titanium. However, the efficiency must be weighed against complex water matrices and of higher pH values, before evaluating its practicality compared to other AOPs.	[25]
Plasma‐induced degradation	NPs Type: Commercial PS (200 nm) [NPs]: 30 mg L^−1^ Voltage = 19 kV	Information on degradation efficiency is not available.	Surface roughening and oxidation were observed. High toxicity of degradation by‐products toward human alveolar cells was noted.	[7]
Phototransformation	NPs Type: Synthesized ^14^C‐radioactively labeled PS (230 nm) [NPs]: 100 m L^−1^ Reaction time: 48 h Light source: UV lamp (emission at 254 nm, 0.7 mW cm^−2^)	Mineralization: 17.1%	UV exposure induced oxidative chain scissions; however, mineralization remained limited, and no morphological changes were observed.	[73]
Photocatalysis	NPs Type: Commercial PS (50‐100 nm) [NPs]: 5 mg L^−1^ Reaction time: 360 min Light source: low‐pressure mercury UV lamp (6 W and 254 nm)	• With UV/NaClO:Turbidity decrease: 78.20%Mineralization: 7%• With UV/PMS:Turbidity decrease: 94.3% Mineralization: 63.90%	PMS outperformed NaClO, achieving higher turbidity reduction and mineralization. SEM confirmed morphology alteration with PMS, while NaClO‐treated particles remained intact. Toxicity was significantly higher for PMS‐treated samples.	[74]
	NPs Type: Synthesized PS (318 nm) [NPs]: 0.9% (w v^−1)^ ^c)^ Reaction time: 50 h Semiconductor: TiO_2_ Immersed area: 1 cm^2^ UV lamp (0.021 mW cm^−2^)	Turbidity decrease:16.2% With TiO_2_‐barrier With TiO_2_‐nanotubular:19.7% With TiO_2_‐ mixed: 23.5%	TiO₂ in various configurations contributed to turbidity reduction with varying effectiveness. Further investigation is needed to optimize UV dependency, enhance mineralization, and reduce toxicity.	[27]
	NPs Type: Polymethylmethacrylate (105 nm) [NPs]: 300 mg L^−1^ pH 6.30 Reaction time: 7 h Semiconductor: TiO_2_– P25/β‐ SiC foams Light source: UVA lamp (15 W, emission at 354 nm, 112 W m^−2^)	Mineralization: 50%	Immobilized TiO₂ photocatalysts showed strong mineralization potential under UVA irradiation. However, the higher effectiveness at low pH value (6.3), may limit practical applications in water treatment due to the need for pH adjustment.	[29]
	NPs Type: Synthesized PS (350 nm) [NPs]: 5 mg mL^−1^ pH: 11.00 Reaction time: 50 h Semiconductor: Immobilized copper oxide Immersed Area: 2.6 cm^2^ Light source: LED (50 W, emission at 400–800 nm, 5.3 mW cm^−2^)	Mineralization: 15% Degradation: 23.5%	FTIR and GC‐MS confirmed the oxidation and breakdown of NPs, but mineralization remained relatively low.	[28]
	NPs Type: PS (140 nm and 300 nm) [NPs]: 100 mg L^−1^ Semiconductor: TiO_2_‐P25 Light source: UV‐A lamp (Philips 24 W/10/4 P lamps, 365 nm, 60 W m^2^)	Information on degradation efficiency is not available.	NP size reduction and shape modification was observed. FTIR showed oxidation, with distinct carbonyl and peroxyl index trends suggesting different reaction pathways which should be further investigated.	[75]
	NPs Type: PS 140 nm and 508 nm [NPs]: 20 mg L^−1^ Semiconductor: TiO_2_‐P25 fixed on β‐SiC foam Light source: 4 UV‐A lamps (Philips T5 15 W 10 Actinic BL, 365 nm, 110 W m^2^)	• Using 140 nm NPs Mineralization: 80% • Using 508 nm NPs Mineralization: 68%	Larger particles showed lower mineralization, indicating size‐dependent efficiency. pH reduction during degradation was considered as an indication for carboxylic acid formation. Py‐GC/MS correlated well with TOC values, supporting its effective use in degradation monitoring.	[75]
	NPs Type: Top‐down chemical fabrication of PET NPs (111 ± 51 nm) from commercial bottles [NPs]: 100 mg L^−1^ pH 3 Reaction time: 5 h Catalyst: 0.125 g/L immobilized TiO_2_/MOF Immersed Area: Held on a fine mesh stainless steel basket (4.2 × 4.2 × 4.4 cm) Light source: Xenon lamp (Wavelengths consistent with sunlight, 30 W m^−2^)	Turbidity reduction: 54.6%	Turbidity reduction was achieved under simulated solar irradiation and FTIR confirmed oxidation of the NPs, at a very low pH value. The lack of data on by‐products and mineralization highlights the need for further investigation.	[76]
	NPs Type: Commercial PS (100 nm) [NPs]: 100 mg L^−1^ pH 7 Reaction time: 15 h Catalyst: 0.31 g/L SnO_2_/g‐C_3_N_4_ immobilized on poly(vinylidene fluoride‐co‐hexafluoropropylene) (3 × 3 cm^2^) Light source: Xenon lamp (Wavelengths consistent with sunlight, 100 mW cm^−2^)	Mineralization: 46%	Immobilized photocatalysts underwent surface erosion and NP fragmentation under simulated solar irradiation. FTIR/XPS confirmed oxidation, while moderate mineralization suggests incomplete breakdown. Further studies are needed to identify by‐products and the degradation pathway.	[69]
Photo‐Fenton	NPs Type: Commercial PS (140 nm) [NPs]: 20 mg L^−1^ [Fe^3+^]_0_ = 1 mg L^−1^ [H_2_O_2_]_0_ = 130 g L^−1^ pH_0_ = 3 Reaction time: 40 min Light source: Medium pressure Hg lamp (150 W, 250 and 600 nm, 200 W m^−2^)	Mineralization: 100% Turbidity decrease: 100%	Complete mineralization was achieved. However, the reliance on low pH and the use of a homogeneous catalyst (Fe^3^⁺), which complicates its recovery from the reaction media, require further investigation for scalability.	[77]
Photo‐Fenton	NPs Type: Commercial PS (215 nm) [NPs]: 25 mg L^−1^ [H_2_O_2_] = 0.5 mol L^−1^ [Na_2_SO_4_] = 0.05 mol L^−1^ pH 2 Flow rate: 50 µL min^−1^ Catalyst: Immobilized MOF‐derived porous Fe_2_O_3_ Immersed Area: 2.5 cm^2^ Light source: Xenon lamp (Wavelengths consistent with sunlight, 120 mW cm^−2^) Bias voltage: 1.2 V	Degradation: 80%	Efficient degradation was achieved under simulated solar irradiation. However, the need for highly acidic conditions, and the potential by‐product toxicity, and electrode stability concerns must be addressed for practical applications.	[68]

a) This group synthesized metal‐labeled PS NPs.

^b)^ Mineralization is monitored with TOC analysis.

^c)^ The solution concentration was expressed as 0.9% (w/v), but the units (g L^−1^ or mg L^−1^) were not specified.

#### 3.1.1 Chemical Oxidation

Among the chemical oxidation processes, ozonation is considered a robust AOP for the remediation of plastic particles in contaminated water.^[^ ^78^, ^79^ ^]^ The reactive agent,O_3_, can attack electron‐rich components such as activated aromatic rings, amines, and double bonds, resulting in ring opening and converting the complex compounds to smaller molecules. In addition, the ^•^OH produced during the ozonation process contributes significantly to oxidizing toxic water pollutants.^[^ ^80^, ^81^ ^]^ Notably, pioneering studies have illustrated that ozonation is a valuable alternative for removing plastic fragments, proving that NPs break down into smaller molecules and gradually mineralize (Table 2).^[^ ^26^, ^70^, ^71^, ^72^ ^]^

This can be attributed to three main reasons: i) O_3_ introduces oxygen to the surface of NPs, enhancing the hydrophilicity and oxidative degradation; ii) the unsaturated and saturated bonds can be attacked directly by O_3_ or indirectly by ROS to open the aromatic rings and produce simple by‐products;^[^ ^26^ ^]^ iii) the produced by‐products can undergo further reactions with the available reactive oxygen radical species, and convert to nontoxic compounds.

**Figure 3:**
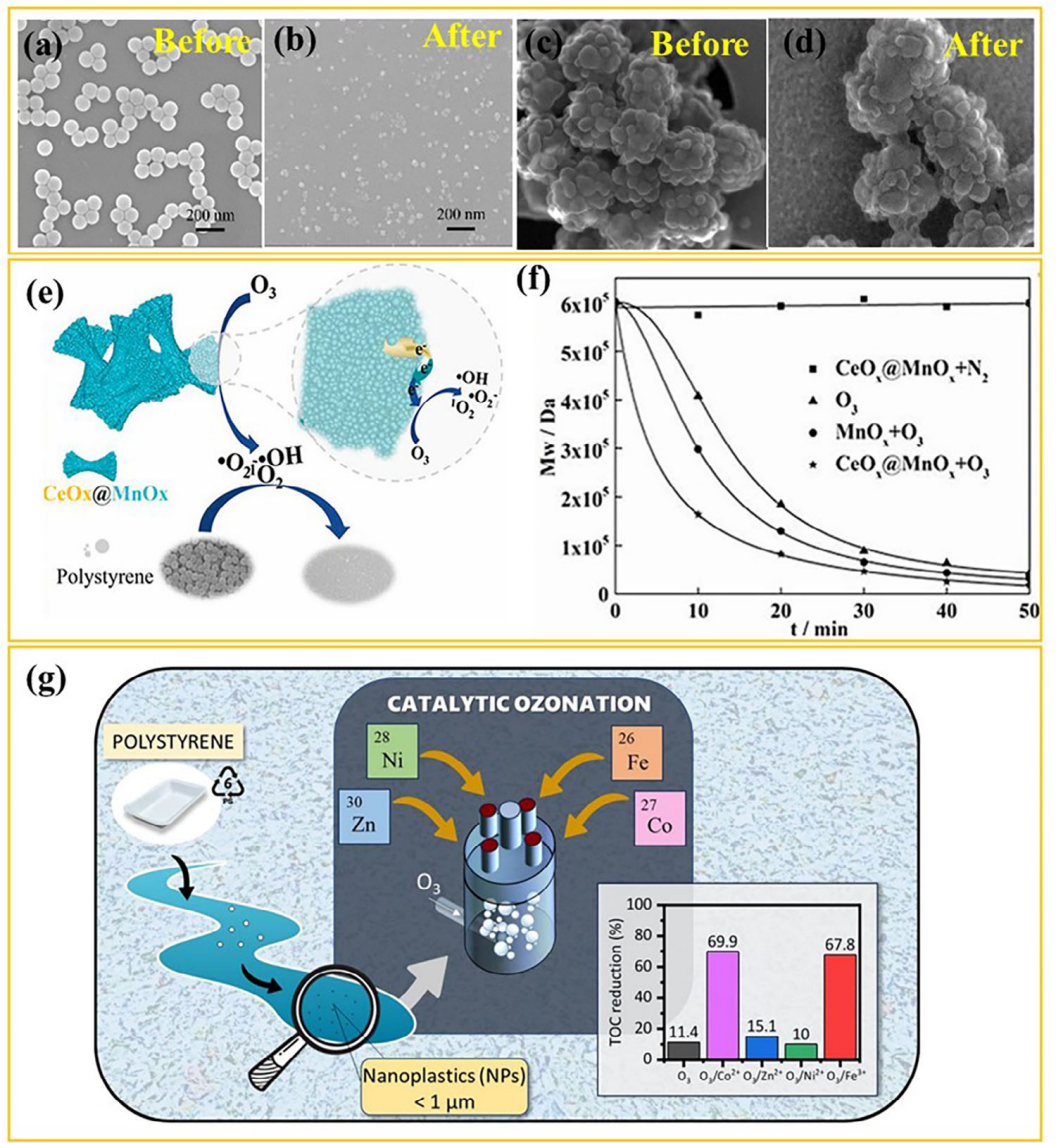
SEM images of PS NPs before a) and after b) 240 min of ozonation. Adapted with permission.^[^ ^26^ ^]^ Copyright 2022, Elsevier. Secondary electron images of PS NPs before c) and after d) ozonation. Adapted under the terms of the CC BY 4.0 license.^[^ ^70^ ^]^ Copyright 2022, Pulido‐Reyes et al. Schematic illustration of the catalytic ozonation process and its efficiency in removing PS NPs using e,f) CeO_x_@MnO_x_ (reproduced with permission.^[^ ^72^ ^]^ Copyright 2023, Elsevier), and g) Co^2^⁺ as catalyst (reproduced under the terms of the CC BY 4.0 license.^[^ ^71^ ^]^ Copyright 2024, Nieto‐Sandoval et al.).

The efficiency of single ozonation in degrading NPs has been recently evaluated using various analytical techniques to assess molecular weight reduction, chemical transformations, and by‐product formation. In particular, Li et al.^[^ ^26^ ^]^ studied the ozonation and chlorination of PS NPs (100 nm) in pure water, revealing that ozonation led to a 42.7% mineralization of the plastic particles after 240 min of treatment. The degradation process was closely monitored using gel permeation chromatography (GPC), which tracked the reduction in molecular weight over time, while SEM confirmed significant size reduction (**Figure** **3**a,b). Additionally, FTIR and XPS revealed the formation of oxygen‐containing functional groups, such as carboxyl, alkyl hydroperoxide, and ketone groups, indicating oxidation‐induced modifications. Pyr‐GC/MS provided further insights into the reaction pathway by detecting seven oxidation by‐products. These findings collectively demonstrate that ozonation effectively breaks down NPs into smaller molecules, although limited mineralization was achieved.

Conversely, Pulido‐Reyes et al.^[^ ^70^ ^]^ investigated the ozonation of Pd‐labeled PS NPs (164–215 nm) in a more environmentally relevant, lake water, setting. Unlike the results of Li et al., no significant morphological changes in NPs were observed via SEM (Figure 3c,d) and dynamic DLS after 45 min of treatment, suggesting that fragmentation and size reduction were negligible. However, zeta potential measurements indicated a decrease in surface charge with increasing ozone dose, hinting at potential surface modifications. This disparity in outcomes between the two studies highlights the crucial role of water matrix composition. While ozonation efficiently degraded NPs in pure water, its efficacy was significantly reduced in the presence of natural organic matter and other competing species naturally present in lake water.

Despite its ability to introduce oxygen functional groups and reduce the molecular weight of NPs, single ozonation faces critical limitations, particularly in real environmental conditions. Its efficiency is highly dependent on factors such as ozone dose, reaction time, and the composition of the surrounding water matrix. In the presence of natural organic matter, competing ions, or other contaminants, ozone's reactivity can be significantly reduced, leading to incomplete degradation and the accumulation of partially oxidized by‐products. This raises concerns about secondary pollution, as smaller, oxygenated plastic fragments may persist in the environment with unknown long‐term effects. Moreover, as demonstrated by Pulido‐Reyes et al.,^[^ ^70^ ^]^ ozonation alone may be insufficient to break down NPs in complex water systems, highlighting the need for more effective treatment strategies.

Given the limitations of single ozonation, catalytic ozonation has been explored to enhance NPs degradation efficiency. This process involves the use of catalysts (e.g., metal oxides, transition metal ions) that accelerate ozone decomposition into highly reactive species, including ^•^OH, ^•^O₂⁻, and ¹O₂. These species facilitate deeper oxidation, ensuring more effective breakdown and mineralization of NPs. Recent studies have demonstrated the superiority of catalytic ozonation over conventional ozonation. Li et al.^[^ ^72^ ^]^ developed a core–shell CeO_x_@MnO_x_ catalyst, which significantly improved the effectiveness of the ozonation process. As depicted in Figure 3e,f, compared to conventional ozonation, which reduced by 31.67% the PS NPs molecular weight within the first 10 min of treatment, catalytic ozonation using MnO_x_ achieved a 51.67% reduction, while CeO_x_@MnO_x_ further increased the performance, reaching a decrease of 73.33%. Notably, after 50 min, CeO_x_@MnO_x_ ozonation achieved a 96.70% reduction, far superior to conventional ozonation. This enhancement is attributed to the electron transfer mechanism within the catalyst, which stabilizes the catalytic cycle, preventing deactivation over multiple reaction cycles.

Similarly, as shown in Figure 3g, Nieto‐Sandoval et al.^[^ ^71^ ^]^ investigated the use of metal ion catalysts (Fe^3^⁺, Co^2^⁺, Ni^2^⁺, Zn^2^⁺) to improve the degradation performance of ozonation for the PS NPs degradation. While single ozonation resulted in a 33% reduction in turbidity and only 16% mineralization of PS NPs after 2 h, Co^2^⁺‐catalyzed ozonation nearly doubled the turbidity reduction (65%) and achieved a remarkable 70% mineralization for the same duration of treatment. This was attributed to the generation of additional hydroxyl radicals via the catalytic decomposition of ozone, leading to more effective degradation and mineralization.

While both ozonation and catalytic ozonation have demonstrated their potential in NPs degradation in controlled laboratory environments, their effectiveness in complex water matrices remains limited. To fully assess its viability in large‐scale water treatment applications, future research should focus on optimizing catalysts, understanding the long‐term environmental impact, and ensuring the safe removal or transformation of the degradation by‐products.

#### 3.1.2 Electrochemical Oxidation

Electrochemical oxidation is a promising method for degrading and mineralizing persistent pollutants, including NPs, by generating ROS like ^•^OH and hydrogen peroxide (H_2_O_2_),^[^ ^82^, ^83^ ^]^ and is considered an eco‐friendly and cost‐effective alternative to conventional degradation methods. Due to these advantages, electrochemical oxidation has been applied in various NPs degradation studies, demonstrating significant removal efficiencies under optimized conditions (Table 2).

**Figure 4:**
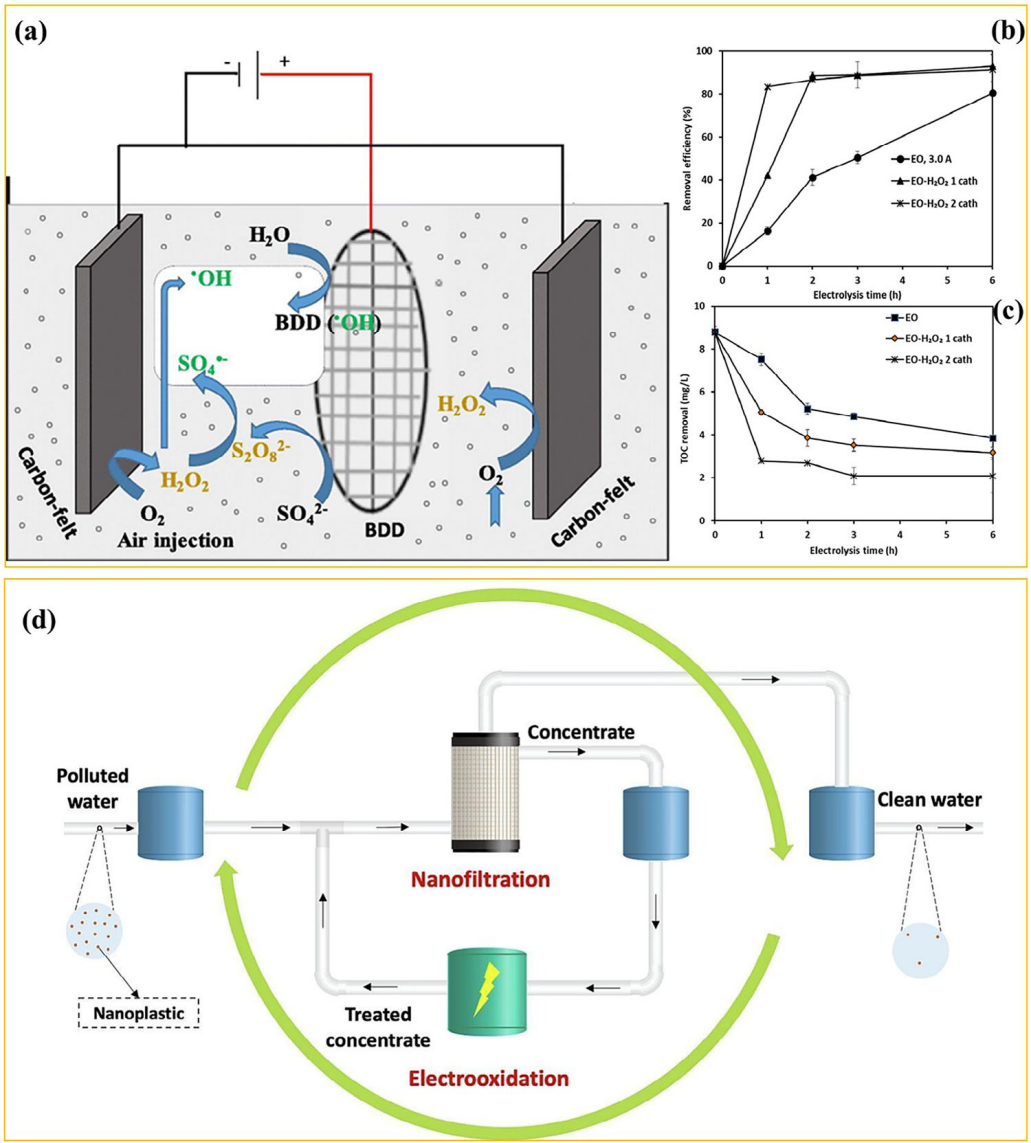
a) Schematic representation of NPs degradation via electrooxidation, b) NPs removal efficiency, and c) mineralization performance. Adapted with permission.^[^ ^25^ ^]^ Copyright 2022, Elsevier. d) Conceptual illustration of a hybrid filtration‐electrooxidation system for completely removing NPs from water. Adapted with permission.^[^ ^84^ ^]^ Copyright 2025, Elsevier.

As demonstrated in **Figure** **4**a, Kiendrebeogo et al.^[^ ^25^ ^]^ used the electro‐oxidation (EO) and electro‐peroxidation (EO‐H_2_O_2_) methods to degrade 100 nm‐sized PS NPs. Both methods used a boron‐doped diamond (BDD) anode with titanium and carbon felt cathodes. It has been demonstrated that in the presence of Na_2_SO_4_ as the supporting electrolyte, a BDD anode can effectively oxidize sulfate ions to persulfate (S_2_O_8_^2−^). This, in turn, plays a crucial role in the oxidation of organic compounds by generating sulfate radicals (SO_4_^•‐^), thereby facilitating the degradation process. As a result, their findings revealed that in both the EO and EO‐H_2_O_2_ systems, hydroxyl radicals ^•^OH and SO_4_^•‐^ were generated. Furthermore, in the EO‐H_2_O_2_ system, an additional generation of hydroxyl radicals ^•^OH and sulfate radicals SO_4_^•‐^ occurred, attributed to the decomposition of H_2_O_2_ and its direct interaction with the produced persulfate ions S_2_O_8_^2‐^. According to Figure 4b, at the optimal conditions, the further generated reactive species during 380 min of EO‐H_2_O_2_ treatment resulted in NPs degradation efficiency of 86.8% ± 1.8%, as determined through spectrophotometric spectral absorption measurements of C═C bonds of PS at 254 nm. This efficiency was 2.6 times higher than that obtained during the EO process. Moreover, it was confirmed that under optimal conditions, a minor portion of the degraded NPs was mineralized (6.50 ± 0.64%) during the EO‐H_2_O_2_ process (Figure 4c). This highlights that although the oxidative fragmentation is facilitated, only a small fraction of degraded NPs undergo complete mineralization, leaving behind partially oxidized intermediates that may persist in the environment.

On top of this, as confirmed by Feilizadeh et al.,^[^ ^84^ ^]^ the effectiveness of EO declines at concentrations of lower NPs due to mass transfer limitations and reduced interaction between NPs and generated ROS. Specifically, although at high NPs concentrations such as 10, 22.5, and 35 mg L^−1^, EO follows predictable degradation kinetics, the degradation efficiency decreases at lower concentrations (< 2 mg L^−1^). To mitigate this issue, EO is integrated with nanofiltration (NF) for improved NPs remediation performance, also at low NPs concentrations (Figure 4d). In particular, NF is introduced to concentrate the NPs in water, followed by EO for their degradation. This combined process significantly improves the NPs removal efficiency, overcoming the limitations in the performance of standalone EO when low NPs concentrations are involved. Furthermore, the combined NF‐EO process reduces the energy consumption requirements of the EO process.^[^ ^84^ ^]^

This study highlights the potential of hybrid filtration‐electrochemical systems in advancing toward more practical and efficient approaches for NPs treatment. In particular, it underscores how integrating NF mitigates the concentration limitations typically associated with EO, enabling the treatment of samples with NP concentrations closer to environmentally relevant levels. By overcoming this constraint, combining EO with NF or other energy‐efficient methods, such as solar‐assisted electrochemical oxidation, could enhance overall sustainability and reduce operational costs. However, catalyst design and electrode materials advancements could further improve reaction efficiency.

#### 3.1.3 Plasma‐Induced Degradation Processes

Electrical discharge plasma is one of the most promising AOPs, providing reactive oxidizing agents such as potential electrons and ^•^OH in situ via high voltage discharge. The generated reactive compounds can attack the chemical bonds of organic pollutants such as plastics and break them into small, nontoxic molecules.^[^ ^85^, ^86^ ^]^ Therefore, plasma‐based degradation processes offer a fast, chemical‐free approach, making them attractive for environmental remediation.

**Figure 5:**
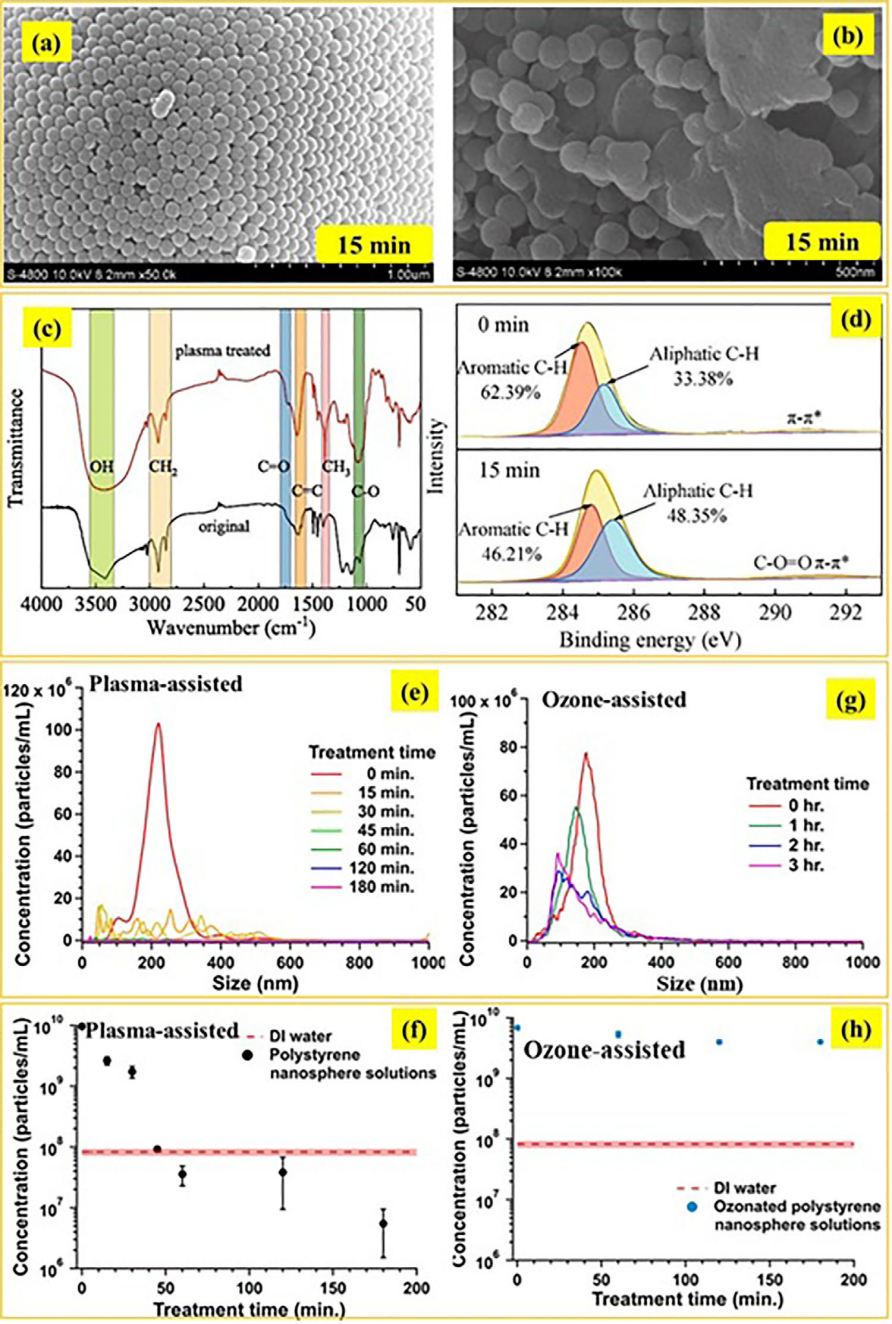
a,b) SEM images of PS NPS before and after 15 min of plasma‐induced treatment. c) FTIR and d) high‐resolution XPS spectra of C1s for pristine and treated NPs. Adapted with permission.^[^ ^7^ ^]^ Copyright 2025, Elsevier. Size distribution of PS NPs at different treatment times e,g) and f,h) concentration reduction over time during plasma‐assisted and ozone‐assisted processes. Adapted with permission.^[^ ^87^ ^]^ Copyright 2024, Royal Society of Chemistry.

Zhou et al.^[^ ^7^ ^]^ evaluated the performance of plasma‐induced degradation of PS NPs (200 nm). According to the morphological analysis (**Figure** **5**a,b), NPs presented some melting, and their surface was roughened after the treatment. The size and zeta potential variations were precisely studied using a nanoparticle potential analyzer. The findings depicted that in a brief time (15 min), the size of all NPs was decreased to 50–150 nm. Moreover, after the treatment, the zeta potential of NPs became more negative due to the hydroxyl and carboxyl groups produced on their surface, as confirmed by FTIR and XPS analyses (Figure 5c,d), suggesting plasma‐induced oxidation effects.

More recently, Winburn et al.^[^ ^87^ ^]^ investigated the plasma‐assisted destruction (PAD) of PS NPs. A comparative study with ozonation highlights the superiority of PAD in NPs remediation. In particular, PAD treatment led to a 99.3% reduction in PS NPs amount within 3 h of treatment (Figure 5e,f), transforming the initially turbid solution into crystal clear water. This degradation was primarily driven by the highly reactive ^•^OH produced in the plasma reactor at a rate of at least 0.2 mm per minute, facilitating fragmentation and further breakdown into short oligomers, particularly dimers. Furthermore, the TOC analysis confirmed a 27.4% mineralization after 3 h. Unlike PAD, which effectively promotes controlled degradation and oligomerization, ozonation failed to achieve NPs degradation and instead promoted fragmentation and the generation of smaller secondary NPs (Figure 5g,h).

As discussed, the degradation of NPs could also result in highly toxic by‐products, leading to secondary environmental pollution. Therefore, toxicity studies of the water where NPs were treated could offer a better insight into evaluating the performance of the degradation process from a different point of view. To this aim, Zhou et al.^[^ ^7^ ^]^ conducted a toxicity study to confirm the efficiency of the PAD process of commercial PS NPs (200 nm). Interestingly, in the presence of treated NPs with a concentration of 30 mg L ^−1^, the growth of *Scenedesmus obliquus*, a green algae species, was not significantly affected by the treated NPs. Notwithstanding, the toxicity of the treated NPs was substantially higher for the human alveolar cells.

These findings underscore the potential of PAD as a robust and efficient strategy for NPs remediation, offering both rapid degradation and advanced oxidation‐driven breakdown beyond mere fragmentation. However, as with other remediation methods discussed in this review, the broader environmental implications remain uncertain, particularly due to the potential formation of reactive by‐products and their unknown biological impact. While PAD effectively reduces NP concentrations and facilitates partial mineralization, similar concerns apply to other advanced oxidation and electrochemical processes, where transformation products may introduce unforeseen environmental risks. Future research should conduct comparative assessments across different methods to evaluate by‐product formation, persistence, and toxicity. In addition, it should focus on refining reactor conditions and minimizing energy demands for PAD. This holistic approach will ensure plasma‐based and advanced treatments maximize degradation efficiency while aligning with long‐term environmental safety standards.

#### 3.1.4 Photocatalytic Process

Photocatalysis is a sustainable, cost‐effective, and widely available method and one of the most extensively used AOPs for water purification. This aligns with the Sustainable Development Goals outlined in the United Nations 2030 Agenda.^[^ ^88^ ^]^ In parallel with the other AOPs, photocatalysis has received significant research attention for the degradation of NPs (Table 2),^[^ ^27^, ^28^, ^29^, ^73^ ^]^ with a significantly higher number of published studies than the other methods (Figure 2).

Photocatalytic degradation occurs through light‐induced reactions, leading to polymer chain scission, surface oxidation, and the generation of ROS. It operates via three primary mechanisms: phototransformation, homogeneous photocatalysis, and heterogeneous photocatalysis. Phototransformation (photolysis) involves the direct breakdown of NPs upon exposure to UV or visible light, either naturally or under controlled conditions. Homogeneous photocatalysis enhances this process by introducing light‐activated oxidants such as H₂O₂, PMS, or chloride ions, which generate additional radicals to accelerate degradation. Heterogeneous photocatalysis, in contrast, relies on solid semiconductors (e.g., TiO₂, ZnO, MOFs) that act as catalysts under irradiation, promoting redox reactions and the generation of electron–hole pairs (e⁻/h⁺), leading to ROS formation. While each mechanism has distinct advantages and limitations, their effectiveness varies depending on reaction conditions, catalyst properties, and pollutant characteristics, as explored in the following sections.

**Figure 6:**
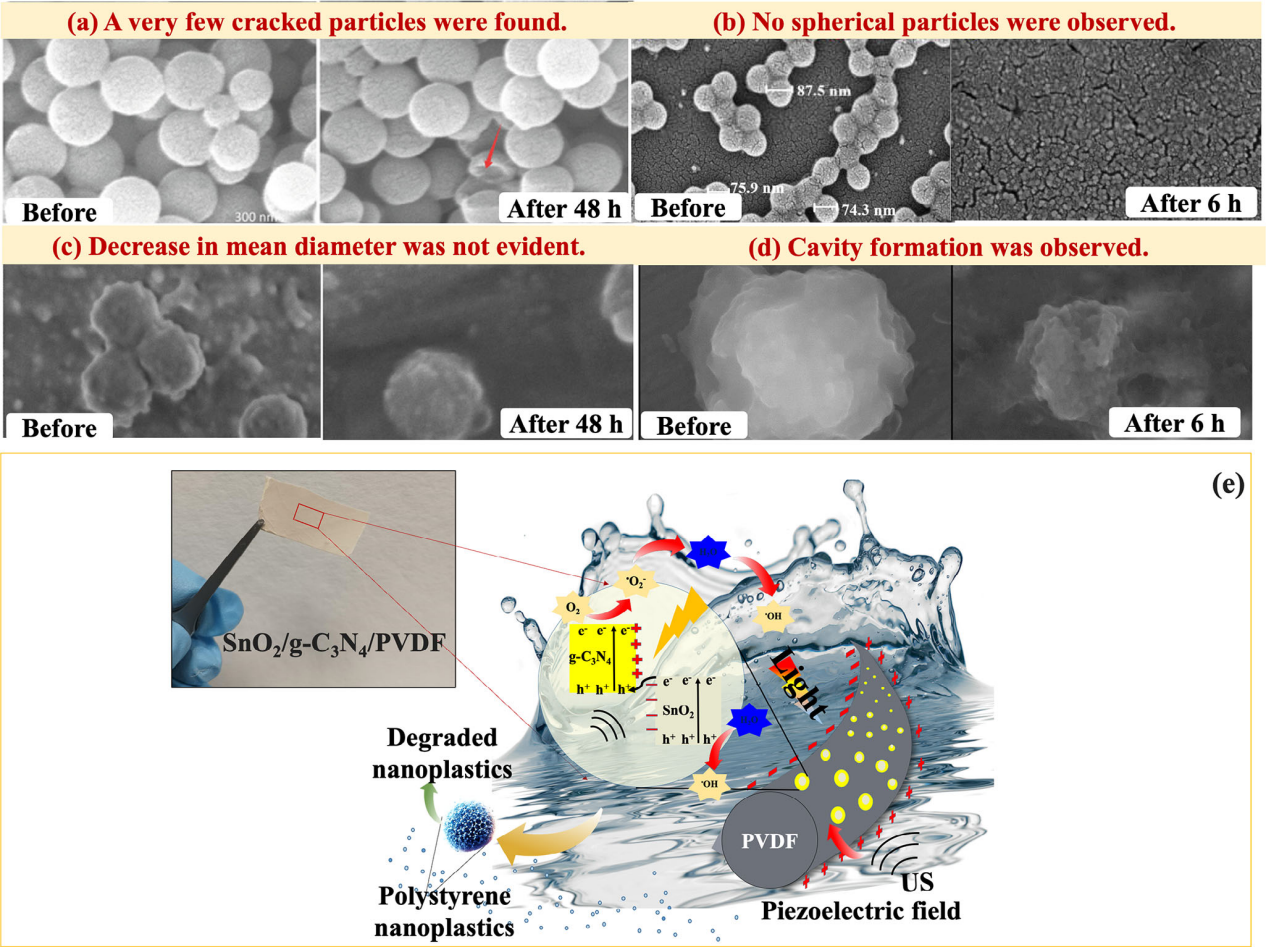
Electron microscopy images of pure and degraded PS NPs during photo‐based processes. a) UV irradiation (adapted with permission.^[^ ^73^ ^]^ Copyright 2019, Royal Society of Chemistry), b) homogenous photocatalytic process (Adapted under the terms of the CC BY 4.0 license.^[^ ^74^ ^]^ Copyright 2023, Cai et al.), and c,d) heterogeneous photocatalytic processes (adapted with permissions.^[^ ^75^ ^]^ Copyright 2022, Elsevier and^[^ ^76^ ^]^ Copyright 2023, Elsevier). Overview of heterogeneous photocatalytic systems used to degrade NPs. e) SnO_2_/g‐C_3_N_4_/PVDF‐HFP composite, along with its piezo‐photocatalytic mechanism. Adapted under the terms of the CC BY 4.0 license.^[^ ^69^ ^]^ Copyright 2024, Fazli et al.

When plastics are exposed to UV light, the absorbed energy triggers radical formation, polymer chain cleavage, and oxidation reactions, leading to NPs degradation and by‐product generation. Tian et al.^[^ ^73^ ^]^ synthesized ¹⁴C‐labeled PS NPs (230 nm) and studied their phototransformation under UV irradiation (254 nm, 0.7 mW cm⁻^2^) in water. After 48 h, 17.1% mineralization was achieved, and minor hydrophilic oxidation by‐products were released, which continued to degrade under prolonged irradiation. However, SEM analysis (**Figure** **6**a) revealed that UV irradiation alone was insufficient to induce significant morphological changes, highlighting the limited efficiency of direct photolysis. Despite being a naturally occurring process, phototransformation often leads to the formation of oxygenated polymer fragments rather than full mineralization. The absence of additional reactive species limits oxidation efficiency, necessitating external photocatalysts or oxidants to enhance degradation.

Given the slow and surface‐limited degradation observed in phototransformation, researchers have explored homogeneous photocatalysis, which enhances NPs breakdown by introducing external oxidants that generate reactive radicals under light irradiation. For instance, Cai et al.^[^ ^74^ ^]^ investigated the role of NaClO and PMS under UV irradiation, generating additional ^•^OH, SO₄^•⁻^, and chlorine radicals (^•^Cl) to facilitate PS NPs degradation. Their findings demonstrated that UV/PMS exhibited superior performance, achieving 94.30% turbidity removal and 63.90% mineralization within 360 min of treatment, compared to 78.20% turbidity removal and only 7% mineralization in the UV/NaClO system. SEM images (Figure 6b) confirmed the loss of spherical NPs after the UV/PMS. However, a significant concern in homogeneous photocatalysis is the formation of toxic by‐products. Toxicity assays revealed that degraded NPs exhibited significantly higher toxicity (98.19% inhibition on luminescent bacteria) compared to untreated NPs (1.98%), suggesting that incomplete mineralization may produce environmentally hazardous components.

Therefore, although homogeneous photocatalysis significantly enhances NPs degradation efficiency, its dependency on external oxidants poses challenges such as secondary pollution, reagent consumption, and reaction control. This has increased interest in heterogeneous photocatalysis, where semiconductor materials act as photoactive catalysts, enabling continuous degradation without additional chemical reagents. Heterogeneous photocatalysis has been widely explored for NPs degradation by employing various semiconductor‐based materials with tailored structures to enhance catalytic efficiency (e.g., TiO_2_,^[^ ^27^ ^]^ Cu_x_O,^[^ ^28^ ^]^ and MOF‐derived porous α‐Fe_2_O_3_^[^ ^89^ ^]^).

TiO₂‐based photocatalysts have demonstrated high efficiency in degrading various polymeric NPs, particularly when optimized for enhancing the surface area and electron transfer dynamics. As shown in the Figure 6c,d, the SEM images reveal the progressive degradation of PS NPs under UV exposure, showing a transition from smooth, spherical particles to fragmented, irregular debris as oxidation advances.^[^ ^76^, ^77^ ^]^ In one study, TiO₂‐P25 was employed under UV‐A irradiation (60 W m^2^) to degrade PS NPs (140 and 300 nm, 100 mg L⁻¹), where FTIR analysis confirmed oxidation through carbonyl and peroxyl formation. However, quantitative mineralization data were not reported. In contrast, a similar system using TiO₂‐P25 immobilized on β‐SiC foam under higher UV‐A intensity (110 W m^2^) achieved significant mineralization, reaching 80% for 140 nm NPs and 68% for 508 nm NPs, highlighting the size‐dependent efficiency of the process. These findings emphasize that the effectiveness of TiO₂‐based photocatalysis depends not only on the photocatalyst type but also on NP characteristics and reaction conditions.

Despite advances in the heterogeneous photocatalytic degradation of NPs, the reported systems predominantly rely on UV light sources, which cover only a small portion of sunlight. Therefore, further studies are needed for a sustainable transit toward effective NPs remediation without the exclusive need for UV light. In this regard, Acuña‐Bedoya et al.^[^ ^28^ ^]^ developed a photocatalyst active under sunlight irradiation composed of immobilized copper oxide semiconductors grown through the anodizing process in NH_4_F media. They studied the degradation of PS NPs under visible light irradiation (Table 2). The reaction process results in a CI increase from 0.70 to 59.75 after 50 h, which, together with the GC/MS analysis, proves the formation of carbonyl groups in the structure of NPs. The degradation process was monitored by turbidity measurements converted to concentration using a calibration curve, while TOC analysis assessed mineralization. According to the results, 23.5% and 15% of NPs degradation and mineralization were observed.

Composite semiconductor systems have also been developed to enhance solar light absorption and charge transfer efficiency. One promising approach involves TiO₂‐based metal–organic frameworks (MOFs), where Fe(III)‐linked MOFs (MIL‐100(Fe)) were incorporated to boost TiO₂ photocatalytic performance.^[^ ^76^ ^]^ This hybrid structure facilitates photoexcited electron transfer to Fe clusters, improving light utilization and overall catalytic efficiency. Immobilized on porous perlite granules, the TiO₂/MOF system demonstrates improved PET NPs degradation compared to the performance of pure TiO₂ after 5 h of treatment. In particular, the CI increased from 0.96 to 0.99, while the turbidity reduction ratio, calculated as the final turbidity relative to the initial turbidity, was 0.454 for the composite, compared to 0.539 for the pure catalyst. Furthermore, the system's optimal performance at pH 3 poses challenges for real‐world applications, as extreme pH conditions could destabilize the photocatalyst and promote metal leaching, potentially transitioning the system from heterogeneous to homogeneous photocatalysis.

A significant challenge in the photocatalytic degradation of NPs lies in catalyst stability and characterization reliability. Allé et al.^[^ ^75^ ^]^ investigated the effectiveness of powder‐based versus immobilized TiO₂ systems, revealing that powder‐based TiO₂ hindered analytical monitoring of NPs degradation due to interference with TOC, UV–vis, and Py‐GC/MS analyses. Immobilized TiO₂ on β‐SiC foams enabled more reliable tracking of NPs degradation using FE‐SEM, TEM, and DLS, suggesting that immobilization, despite reducing catalyst‐NP interaction, remains the preferred strategy for ensuring accurate degradation assessments. TiO₂–P25/β‐SiC foams under 7 h of UVA irradiation achieved 50% mineralization of polymethylmethacrylate (PMMA) NPs, suggesting their potential for scalable remediation applications. A detailed assessment of experimental conditions revealed that lower flow rates in the photocatalytic reactor resulted in higher degradation efficiency, while increased UV intensity accelerated reaction kinetics.

Moreover, hybrid piezo‐photocatalytic systems have emerged as a strategy to further enhance NPs degradation under natural conditions. The Z‐scheme charge transfer mechanism extends the photocatalytic activity of SnO₂/g‐C₃N₄ composites beyond the UV range into the visible spectrum. These composites, immobilized on electrospun polyvinylidene fluoride‐hexafluoropropylene (PVDF‐HFP) membranes, harness the mechanical energy for further catalytic activation. The inherent piezoelectric properties of PVDF‐HFP play a crucial role in this process by generating an internal electric field under mechanical deformation induced by ultrasounds. This effect facilitates charge carrier separation and significantly reduces the recombination rate of electron‐hole pairs, thereby enhancing the generation of Z‐scheme SnO₂/g‐C₃N₄ photocatalyst, leading to the production of more ROS and improving NPs oxidation efficiency (Figure 6e).^[^ ^69^ ^]^ In degradation studies, this system achieved 46% mineralization within 15 h, with SEM, nanoparticle tracking analysis (NTA), and FTIR confirming significant NPs fragmentation and chemical transformation.

Overall, photocatalytic processes represent a significant advancement in NPs remediation, leveraging renewable energy sources and expanding beyond UV‐dependent systems. While studies demonstrate substantial morphological transformations—including size reduction, surface oxidation, and fragmentation—current approaches often fail to complete mineralization, leaving behind smaller particles and potentially more toxic by‐products. The formation of oxygenated polymer fragments raises environmental concerns, as these intermediates may exhibit higher bioavailability and reactivity than the original NPs. Furthermore, photocatalytic efficiency is highly dependent on reaction conditions, catalyst stability, and the complexity of real‐world environmental matrices, posing challenges for large‐scale applications.

To establish photocatalysis as a viable and scalable NPs degradation strategy, future research must focus on integrating toxicity assessments, optimizing catalyst performance under variable conditions, and ensuring long‐term stability without secondary pollution risks. Combining photocatalysis with other AOPs or hybrid treatment systems may further improve mineralization rates and environmental safety. Addressing these challenges is critical to bridging the gap between laboratory findings and real‐world applications, ensuring that photocatalytic technologies contribute meaningfully to the sustainable and effective removal of NPs from aquatic environments.

#### 3.1.5 Fenton

The Fenton reaction is a well‐established AOP that relies on the reaction between H₂O₂ and ferrous ions (Fe^2^⁺) to generate highly reactive ^•^OH, which can effectively degrade organic pollutants^[^ ^61^ ^]^ (Reaction 1). However, in its conventional form, the Fenton process is limited by the accumulation of Fe^3^⁺, which reduces its efficiency over time. Alternatively, the photo‐Fenton process (UV/H₂O₂/Fe^3^⁺) overcomes this limitation by utilizing UV or visible light to regenerate Fe^2^⁺, thereby increasing the production of hydroxyl radicals. This cyclic regeneration enhances the efficiency of pollutant degradation, making it a promising method for NPs degradation in aqueous environments.

(1) $${\mathrm{Fe}}^{2+}+H_{2}O_{2}+h\nu \;\to {\mathrm{Fe}}^{3+}+2^{•}\mathrm{OH}$$

**Figure 7:**
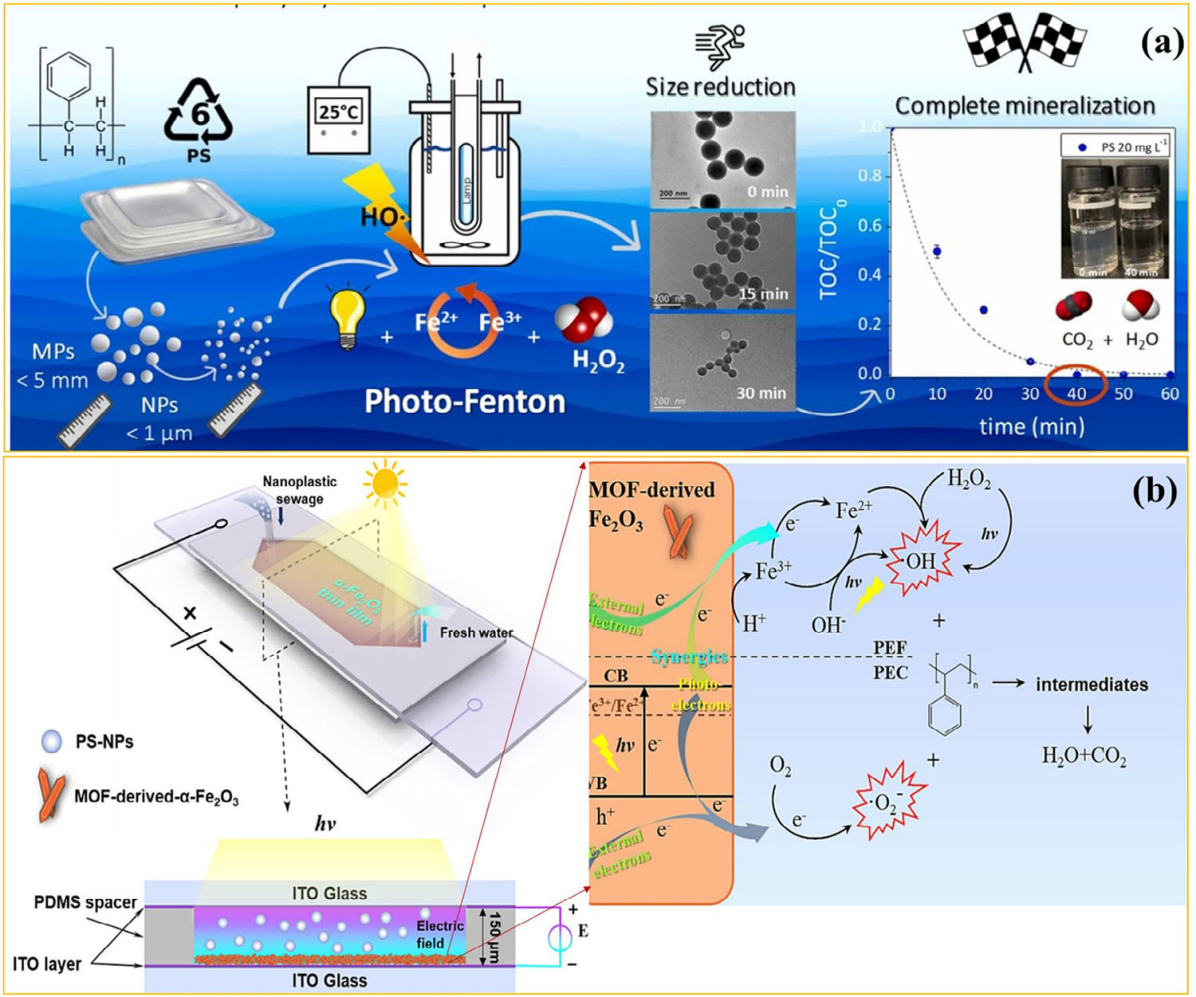
a) Schematic representation of the photo‐Fenton process for PS NPs degradation in water. The process involves Fe^2^⁺‐catalyzed H₂O₂ decomposition to generate ^•^OH under light irradiation, leading to PS NPs oxidation, size reduction, and eventual mineralization into CO₂ and H₂O. The inset images illustrate the progressive fragmentation of PS NPs over time, as observed via TEM, while the TOC reduction graph confirms the near‐complete mineralization within 40 min. Adapted under the terms of the CC BY 4.0 license.^[^ ^77^ ^]^ Copyright 2023, Luca et al. b) Microreactor featuring a MOF‐derived Fe_2_O_3_ film, including a schematic representation of the photoelectro‐Fenton mechanism. Adapted with permissions.^[^ ^68^ ^]^ Copyright 2023, Elsevier.

In particular, as shown in **Figure** **7**a, Luca et al.^[^ ^77^ ^]^ explored the feasibility of degrading NPs based on the Fenton reaction. Under the optimal condition reported in Table 2, the mineralization efficiency followed the trend UV/H_2_O_2_/Fe^3+^ > UV/H_2_O_2_ > H_2_O_2_/Fe^3+^ > photolysis, underscoring the significance of additional ^•^OH production in the degradation of NPs. The turbidity, TOC, and TEM analyses confirmed the thorough degradation and mineralization of PS NPs after 60 min of the photo‐Fenton process (UV/H_2_O_2_/Fe^3+^). In addition, TEM images revealed that the size of PS NPs decreased during the degradation process. Despite the total mineralization observed, the use of UV as the light source and the homogeneous addition of iron (II) sulfate heptahydrate and H_2_O_2_ in the reaction media may limit the applicability of this process for degrading water pollutants, including NPs.

In addition, reactors play a crucial role in the photocatalytic degradation performance. Optofluidic catalytic reactors offer advantages such as a large specific surface area, high mass transfer, and photon utilization efficiency, and have been reported to enhance the removal efficiency of soluble water pollutants.^[^ ^68^ ^]^ In the case of NPs, Chen et al.^[^ ^68^ ^]^ introduced a photoelectron‐Fenton microreactor to degrade PS NPs, as illustrated in Figure 7b. They synthesized a MOF‐derived porous α‐Fe_2_O_3_ thin film and coated it in the reaction chamber to facilitate Fenton reactions. Under low pH conditions, available H_2_O_2_ and leached Fe^2+^ from α‐Fe_2_O_3_ initiated Fenton reactions producing ^•^OH and Fe^3+^.

Additionally, electrons and holes were generated through the light absorption of α‐Fe_2_O_3_. The photogenerated electrons could combine with Fe^3+^ to regenerate Fe^2+^ for further Fenton reactions, leading to the continuous production of ^•^OH for attacking NPs in water. Such a designed photo‐reactor achieved over 80% degradation, monitored through the turbidity reduction of the PS NPs dispersion in water, under 1.2 V bias and 120 mW cm^2^ of simulated solar irradiation. While this is a well‐performing process, no information was provided on the mineralization of potential by‐products. Furthermore, similar to other studies, the reaction conditions do not accurately reflect the complexity of real‐world environmental samples, where various types of environmentally relevant NPs are present in complex organic matrices.

Overall, while the photo‐Fenton and photoelectro‐Fenton processes present effective strategies for NPs degradation, several challenges must be addressed to improve their applicability in real‐world conditions. Specifically, most Fenton‐based processes require more acidic conditions (pH < 3–4), compared to environmental water samples. In addition, while photo‐Fenton regenerates Fe^2^⁺ from Fe^3^⁺, iron accumulation and sludge formation can still occur. Furthermore, most studies monitor NPs degradation via turbidity or TOC, but detailed tracking of degradation intermediates is needed to ensure safe and complete mineralization. Therefore, future studies should focus on enhancing environmental feasibility, improving catalyst stability, and ensuring complete mineralization of NPs residues. Specifically, pH‐neutral or heterogeneous catalysts, active in visible light to harness natural sunlight, and reactor designs with improved Fe‐recycling strategies (e.g., immobilized catalysts) should be explored.

## 4 Current Research Gaps and Perspectives in NPs Remediation Through AOPs

While various AOPs have been explored for NPs degradation, several challenges hinder their scalability and real‐world application. It is essential to address these limitations to bridge the gap between laboratory findings and practical implementation. The following sections explore these overlooked challenges, methodological limitations, and knowledge gaps that must be addressed to develop truly sustainable and effective AOP‐based solutions for NPs degradation. However, the study of NPs degradation through AOPs is still in its early stages, and it is unsurprising if the points mentioned here cover only some of the research gaps in this field. For instance, given that NPs act as insoluble water contaminants, developing efficient reactors and highly effective immobilized heterogeneous catalytic systems is imperative. Optimizing reactor configurations to enhance mass transfer and light utilization efficiency can significantly improve degradation performance while reducing operational costs.^[^ ^68^, ^75^ ^]^ Current AOPs often aim to fully degrade NPs into their mineralized forms, which requires chemical additives, UV light sources, and extended reaction times. Hence, they may inadvertently lead to secondary water pollution and increased energy consumption. Moreover, to facilitate reproducibility and comparability across studies, establishing standardized experimental protocols that better simulate real wastewater conditions, such as pH (typically 6–8), the presence of dissolved organic matter, ionic strength, and competing contaminants, is essential for evaluating the effectiveness of different AOPs in NP degradation.

### 4.1 Overlooked Challenges Stemming from the Complex Environment

Most studies on the NPs’ environmental fate and their remediation approaches are performed using monodispersed artificial nanobeads, mostly PS NPs, at high concentrations far from the environmentally relevant ones. However, these conditions do not usually represent the various types of NPs present in the environment and their expected concentration and, therefore, do not reflect real‐world exposure. These plastic nanostructures mainly possess few defects and homogeneous chemical composition, substantially different from the NPs dispersed in the environment. These have irregular shapes and complex surface chemistry due to their experienced degradation. This raises questions about the accuracy and reliability of the obtained results.^[^ ^54^ ^]^ Hence, for a more accurate assessment of NPs' environmental implications, it is essential to use more environmentally relevant media or gather them directly from their natural surroundings.

Environmental concentrations of NPs are estimated to be extremely low (1 pg L^−1^ – 15 µg L^−1^ for NPs of 50nm^[^ ^90^ ^]^), often embedded within complex organic matrices. These factors complicate their detection, collection, and quantification. Before being characterized or quantified, liquid samples should be processed with various pretreatment steps to separate the NPs from their complex organic matrices.^[^ ^54^, ^91^ ^]^ On the other hand, due to the deficient environmental concentrations of the NPs, much lower than the detection limit of some characterization techniques, pre‐concentration methods such as ultrafiltration, centrifugation, cloud point extraction, and freeze‐drying should be applied before the proper characterization.^[^ ^92^, ^93^, ^94^ ^]^

Confronted with the complexities of collecting and processing environmental samples, scientists have sought alternative approaches to mimic environmentally relevant NPs through the mechanical and photocatalytic degradation of larger plastic fragments. Employing various top‐down methodologies, such as laser ablation^[^ ^95^ ^]^ and ball milling,^[^ ^96^ ^]^ these efforts have resulted in the fabrication of NPs with properties closely resembling those found in natural environments. However, despite this achievement, such approaches have predominantly been utilized for toxicological evaluations and have seen limited integration into NPs degradation through AOPs.

Moreover, wastewater is replete with diverse organic and inorganic compounds, which can impede the efficiency of water pollutant degradation processes.^[^ ^97^ ^]^ NPs are solid water pollutants characterized by their small size and high surface area. Hence, in real‐world environmental samples, NPs are rarely pristine; they often harbor various organic and inorganic components sorbed on their surface.^[^ ^98^ ^]^ In addition, environmental samples typically contain different NPs types. These factors pose significant constraints on the feasibility of degradation processes. While the effects of environmental factors like pH variations, water complexity, and the presence of ions and organic compounds on degrading water‐soluble pollutants have been studied, a significant gap exists in understanding how these factors affect NPs degradation.^[^ ^97^, ^99^ ^]^

Finally, different polymer types and NPs in different media may undergo distinct degradation pathways, leading to the formation of varying intermediate by‐products, some of which could pose potential environmental and toxicological risks. However, this aspect is partially explored in current studies. Future research should focus on examining the degradation behaviors of diverse polymeric NPs under AOP treatments to understand the nature and persistence of by‐products better and assess their potential environmental hazards. Addressing these knowledge gaps is essential for accurately assessing AOPs’ effectiveness under realistic conditions.

### 4.2 Limitations in Characterizing NPs After AOPs

**Figure 8:**
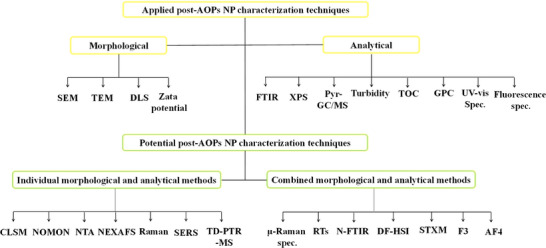
Overview of characterization methods utilized and potentially beneficial for analyzing degraded NPs through AOPs.

Regrettably, there is still a lack of reliable analytical techniques that are both straightforward and sensitive enough to detect individual plastic particles (< 1 µm) from complex mixtures of various polymers, additives, and degradation products. This makes their analysis even more challenging. Most of the so‐far reported characterization techniques for NPs are presented in **Figure** **8**. Specifically, SEM, TEM, dynamic light scattering (DLS), zeta potential, FTIR, and XPS are widely used techniques for studying the morphology, size distribution, shape, surface charge, and chemical composition of the degraded NPs.^[^ ^100^ ^]^ However, such analyses can be challenging due to their low resolution and high detection limit, which demand high NPs concentrations, but also due to the NPs agglomeration and low particle size, limiting their applicability in some instances.^[^ ^36^, ^52^ ^]^ Due to these constraints, advanced technological solutions should be adopted to study the degradation of NPs.

Recent advancements in NPs characterization, rarely considered for AOPs, could prove invaluable for addressing such challenges in future studies. For instance, confocal laser scanning microscopy (CLSM) offers a non‐destructive approach to analyzing the size, shape, and distribution of NPs in complex matrices.^[^ ^101^ ^]^ Advanced optical nanoscopes such as the Nanoro‐M uses a super‐resolution amplifying lens to enhance resolution and image quality and enables detailed characterization of NPs in water.^[^ ^52^ ^]^ NTA provides valuable insights into size distribution, shape, and concentration by analyzing individual particles.^[^ ^102^ ^]^ In addition, near‐edge X‐ray absorption exemplary spectra (NEXAFS) are a powerful tool that can be used to investigate the chemical and structural properties of plastic particles that are less than 100 nm in size.^[^ ^103^ ^]^

In contrast to FTIR, which is more appropriate for larger particles, with higher particle concentrations and polar functional groups, Raman spectroscopy, including surface‐enhanced Raman spectroscopy (SERS), provides chemical fingerprint identification with exceptional sensitivity, allowing for the analysis of NPs at low concentrations.^[^ ^104^, ^105^ ^]^ Furthermore, to analyze the formation of various intermediates during the degradation process, Pyr‐GC/MS and fluorescent spectroscopy were employed. These methods are sensitive to impurities and possess high detection limits. Hence, pretreatment and concentration of the NPs samples are necessary.^[^ ^106^ ^]^ However, a recent study proved that the thermal desorption‐proton transfer reaction‐mass spectrometry (TD‐PTR‐MS) was appropriate for the characterization of NPs at lower concentrations and in natural environments.^[^ ^107^ ^]^

In addition to these methods, established combined techniques can further enhance the simultaneous morphological and chemical analysis, increasing the accuracy of results when studying the degradation of NPs in real‐world samples.^[^ ^14^, ^15^ ^]^ For instance, Raman spectroscopy can be integrated with microscopy (µ‐Raman spectroscopy) to identify NPs at a minimum size of 1 µm.^[^ ^108^ ^]^ Furthermore, Raman spectroscopy integrated with optical tweezers, known as the Raman tweezers (RTs), can trap and characterize plastics in the range of < 1 µm, even in seawater.^[^ ^106^, ^108^ ^]^ Nano‐FTIR and visible‐near infrared dark‐field hyperspectral imaging (DF‐HSI) combine microscopy with spectroscopy, allowing imaging and chemical identification of various plastic particles with sizes less than 1000 nm.^[^ ^109^, ^110^ ^]^ Scanning transmission X‐ray microscopy (STXM) combines the merits of X‐ray microscopy and NEXAFS. It can be used for the chemical analysis of NPs.^[^ ^103^ ^]^ Separation techniques like field‐flow fractionation (F3) and asymmetric flow field‐flow fractionation (AF4) enable the isolation of particles based on physical characteristics, facilitating comprehensive analysis of particle interactions in complex environments.^[^ ^111^ ^]^

### 4.3 Unexplored Long‐Term Risks of AOP‐Derived Intermediates

As already extensively discussed in this review, AOPs can generate ROS that partially degrade NPs, forming low‐molecular‐weight oligomers, monomers, fragmented NPs with oxidized surfaces, and free radicals. These degradation intermediates may exhibit increased mobility, altered bioavailability, and potential toxicity compared to their parent NPs. Moreover, depending on the reaction medium, some oxidized by‐products may undergo repolymerization or secondary reactions, forming new persistent compounds that can remain in aquatic environments for extended periods. The biological impacts of these intermediates, however, remain largely unexplored. Some oxidative by‐products have been shown to induce cytotoxicity, oxidative stress induction, and potential endocrine‐disrupting effects in aquatic organisms. However, long‐term studies on bioaccumulation, trophic transfer, and chronic exposure risks are still lacking, making it difficult to determine the full environmental implications of AOP‐treated NPs.

To ensure the safe and sustainable application of AOPs for NPs degradation, it is crucial to develop strategies that minimize the formation and persistence of toxic by‐products while assessing their long‐term environmental risks. Optimizing reaction conditions, such as pH, oxidant dosage, irradiation intensity, and catalyst concentration, can enhance degradation efficiency and reduce the accumulation of partially oxidized intermediates. Advances in catalyst design can further improve the selectivity of radical reactions toward complete mineralization. Moreover, hybrid AOP systems promise to improve selectivity toward complete NPs mineralization while reducing the accumulation of toxic polymer fragments. Further research on synergistic AOP mechanisms is needed to refine these hybrid approaches, ensuring more controlled and effective degradation pathways with minimal environmental impact.

Integrating post‐treatment technologies, such as adsorption (e.g., activated carbon, biochar, zeolites) and biological treatments (e.g., microbial degradation, biofiltration), can help capture and further break down residual by‐products before their release into aquatic systems. Additionally, real‐time monitoring techniques, including high‐resolution mass spectrometry and nuclear magnetic resonance, are essential for tracking AOP‐derived intermediates and assessing their persistence in water. Understanding the long‐term ecological risks associated with these by‐products requires standardized ecotoxicity screening and chronic exposure studies to evaluate bioaccumulation, genotoxicity, and ecosystem‐level impacts. By implementing these mitigation strategies and risk assessment frameworks, future research can ensure that AOPs remain a viable and environmentally responsible technology for NPs remediation without introducing new ecological hazards.

## 5 Conclusion

NPs from primary and secondary sources find their way into the water bodies. Even at low concentrations, the detected NPs in drinking water and food led to concerns about their potential hazards. Although various conventional methods were applied to remove NPs from water, their complexity and limited performance restrict their wide application. AOPs are alternative methods that can produce reactive radical species to degrade NPs into small, harmless compounds. Considering the efficiency of these methods, several AOPs were applied to the degradation of plastic particles. As reported, ozonation, electro‐oxidation, electro‐peroxidation, plasma‐induced, and photocatalytic processes showed an effective performance for the degradation of NPs. Nonetheless, considering the slow publication rate in applying AOPs for the in situ degradation of NPs, this emerging field remains in its infancy, necessitating more significant effort to explore robust AOPs with improved efficiency and minimal environmental impact.

Moreover, existing research primarily involves artificial NPs and controlled environmental conditions, highlighting the crucial need for investigations using environmentally realistic NPs samples under potentially hazardous situations. This review reveals that sample preparation for various characterization techniques needs to be more specific, underscoring the importance of providing precise experimental and analytical details. Coordinated efforts are therefore essential to enhance experimental protocols and address the remaining challenges. This collective effort will enhance understanding of NP degradation through AOPs, paving the way for sustainable and effective NPs remediation, even at an industrial scale.

## Conflict of Interest

The authors declare no conflict of interest.

## References

[1] V. Percec , Q. Xiao , Chem 2020, 6, 2855.

[2] Q. Liu , Z. Chen , Y. Chen , F. Yang , W. Yao , Y. Xie , J. Agric. Food Chem. 2021, 69, 10450. 34473500 10.1021/acs.jafc.1c04199

[3] J. Domenech , C. Cortés , L. Vela , R. Marcos , A. Hernández , Biomolecules 2022, 11, 859. 10.3390/biom11060859 PMC8227673 34207836

[4] Global plastic production, Statista , https://www.statista.com/statistics/282732/global‐production‐of‐plastics‐since‐1950/ (accessed: May 2025).

[5] S. Kahlert , C. R. Bening , Resour. Conserv. Recycl. 2022, 181, 106279. 10.1016/j.resconrec.2020.104948 PMC7229953 32427210

[6] The New Plastics Economy: Rethinking the future of plastics , https://www.ellenmacarthurfoundation.org/the‐new‐plastics‐economy‐rethinking‐the‐future‐of‐plastics (accessed: May 2025).

[7] L. Zhou , R. Wang , Y. Liu , et al., Chem. Eng. J. 2022, 433, 134543.

[8] O. S. Alimi , J. Farner Budarz , L. M. Hernandez , N. Tufenkji , Environ. Sci. Technol. 2018, 52, 1704. 29265806 10.1021/acs.est.7b05559

[9] K. L. E. Berry , N. Hall , K. Critchell , K. Chan , B. Bennett , M. Mortimer , P. J. Lewis , Mar. Pollut. – Monit. Manag. Mitig. 2023, 207.

[10] A. A. Mohana , S. M. Farhad , N. Haque , B. K. Pramanik , Chemosphere 2021, 284, 131430. 34323805 10.1016/j.chemosphere.2021.131430

[11] N. B. Hartmann , T. Hüffer , R. C. Thompson , M. Hassellöv , A. Verschoor , A. E. Daugaard , S. Rist , T. Karlsson , N. Brennholt , M. Cole , M. P. Herrling , M. C. Hess , N. P. Ivleva , A. L. Lusher , M. Wagner , Environ. Sci. Technol. 2019, 53, 1039. 30608663 10.1021/acs.est.8b05297

[12] S. M. O'Neill , J. Lawler , Chem. Environ. Eng. 2021, 3, 100091.

[13] J. Domenech , M. de Britto , A. Velázquez , S. Pastor , A. Hernández , R. Marcos , Biomolecules 2021, 11, 1442. 34680075 10.3390/biom11101442 PMC8533059

[14] I. Ali , Q. Cheng , T. Ding , Q. Yiguang , Z. Yuechao , H. Sun , C. Peng , I. Naz , J. Li , J. Liu , J. Clean. Prod. 2021, 313, 127863.

[15] S. M. Patil , N. R. Rane , P. O. Bankole , P. Krishnaiah , Y. Ahn , Y.‐K. Park , K. K. Yadav , M. A. Amin , B.‐H. Jeon , Chem. Eng. J. 2022, 430, 132913.

[16] A. S. I. Abdoul Magid , M.d. S. Islam , Y. Chen , L. Weng , J. Li , J. Ma , Y. Li , Sci. Total Environ. 2021, 784, 147115. 34088021 10.1016/j.scitotenv.2021.147115

[17] Y. Gong , Y. Bai , D. Zhao , Q. Wang , Water Res. 2022, 208, 117884. 34837810 10.1016/j.watres.2021.117884

[18] Y. Zhang , A. Diehl , A. Lewandowski , K. Gopalakrishnan , T. Baker , Sci. Total Environ. 2020, 720, 137383. 32325555 10.1016/j.scitotenv.2020.137383 PMC7241221

[19] Z. Chen , C. Chen , X. Luo , J. Liu , Z. Huang , Appl. Clay Sci. 2021, 213, 106264.

[20] Z. Chen , Z. Huang , J. Liu , E. Wu , Q. Zheng , L. Cui , J. Hazard. Mater. 2021, 406, 124697. 33307450 10.1016/j.jhazmat.2020.124697

[21] Z. Chen , J. Liu , C. Chen , Z. Huang , Chemosphere 2020, 252, 126450. 32222522 10.1016/j.chemosphere.2020.126450

[22] K. Vogel , R. Wei , L. Pfaff , D. Breite , H. Al‐Fathi , C. Ortmann , I. Estrela‐Lopis , T. Venus , A. Schulze , H. Harms , U. T. Bornscheuer , T. Maskow , Sci. Total Environ. 2021, 773, 145111. 33940717 10.1016/j.scitotenv.2021.145111

[23] R. Wang , L. Zhang , B. Chen , X. Zhu , J. Membr. Sci. 2020, 614, 118470.

[24] M. Shen , B. Song , C. Zhou , T. Hu , G. Zeng , Y. Zhang , Sci. Total Environ. 2022, 842, 156723. 35714750 10.1016/j.scitotenv.2022.156723

[25] M. Kiendrebeogo , M. R. Karimi Estahbanati , Y. Ouarda , P. Drogui , R. D. Tyagi , Sci. Total Environ. 2022, 808, 151897. 34826468 10.1016/j.scitotenv.2021.151897

[26] Y. Li , J. Li , J. Ding , Z. Song , B. Yang , C. Zhang , B. Guan , Chem. Eng. J. 2022, 427, 131690.

[27] L. P. Domínguez‐Jaimes , E. I. Cedillo‐González , E. Luévano‐Hipólito , J. D. Acuña‐Bedoya , J. M. Hernández‐López , J. Hazard. Mater. 2021, 413, 125452. 33930967 10.1016/j.jhazmat.2021.125452

[28] J. D. Acuña‐Bedoya , E. Luévano‐Hipólito , E. I. Cedillo‐González , L. P. Domínguez‐Jaimes , A. M. Hurtado , J. M. Hernández‐López , J. Environ. Chem. Eng. 2021, 9, 106208. 10.1016/j.jhazmat.2021.125452 33930967

[29] P. H. Allé , P. Garcia‐Muñoz , K. Adouby , N. Keller , D. Robert , Environ. Chem. Lett. 2020, 19, 1803.

[30] Y. Zhang , F. Cheng , T. Zhang , C. Li , J. Qu , J. Chen , W. J. G. M. Peijnenburg , Environ. Sci. Technol. 2022, 56, 3085. 35174701 10.1021/acs.est.1c07129

[31] D. Kumar , J. K. Biswas , S. I. Mulla , R. Singh , R. Shukla , M. A. Ahanger , G. S. Shekhawat , K. K. Verma , M. W. Siddiqui , C. S. Seth , Plant Physiol. Biochem. 2024, 213, 108795. 38878390 10.1016/j.plaphy.2024.108795

[32] I. Ali , X. Tan , J. Li , C. Peng , P. Wan , I. Naz , Z. Duan , Y. Ruan , Water Res. 2023, 230, 119526. 36577257 10.1016/j.watres.2022.119526

[33] P. D. Sutrisna , L. Riadi , P. C. W. Buana , K. Khoiruddin , R. Boopathy , I. G. Wenten , U. W. R. Siagian , Polym. Degrad. Stab. 2024, 220, 110635.

[34] A. Kundu , N. P. Shetti , S. Basu , K. R. Reddy , M. N. Nadagouda , T. M. Aminabhavi , Chem. Eng. J. Lausanne Switz. 2021, 421, 1996. 10.1016/j.cej.2021.129816 PMC8422880 34504393

[35] B. K. Pramanik , S. K. Pramanik , S. Monira , Chemosphere 2021, 282, 131053. 34098311 10.1016/j.chemosphere.2021.131053

[36] S. Gangadoo , S. Owen , P. Rajapaksha , K. Plaisted , S. Cheeseman , H. Haddara , V. K. Truong , S. T. Ngo , V. V. Vu , D. Cozzolino , A. Elbourne , R. Crawford , K. Latham , J. Chapman , Sci. Total Environ. 2020, 732, 138792. 32442765 10.1016/j.scitotenv.2020.138792

[37] S. Lambert , M. Wagner , Chemosphere 2016, 145, 265. 26688263 10.1016/j.chemosphere.2015.11.078 PMC5250697

[38] S. Prenner , A. Allesch , M. Staudner , M. Rexeis , M. Schwingshackl , M. Huber‐Humer , F. Part , Environ. Pollut. 2021, 290, 118102. 34523518 10.1016/j.envpol.2021.118102

[39] M. Mathissen , V. Scheer , R. Vogt , T. Benter , Atmos. Environ. 2011, 45, 6172.

[40] A. L. Dawson , S. Kawaguchi , C. K. King , K. A. Townsend , R. King , W. M. Huston , S. M. Bengtson Nash , Nat. Commun. 2018, 9, 1001. 29520086 10.1038/s41467-018-03465-9 PMC5843626

[41] L. Peng , D. Fu , H. Qi , C. Q. Lan , H. Yu , C. Ge , Sci. Total Environ. 2020, 698, 134254. 31514025 10.1016/j.scitotenv.2019.134254

[42] W. C. Li , H. F. Tse , L. Fok , Sci. Total Environ. 2016, 566, 333. 27232963 10.1016/j.scitotenv.2016.05.084

[43] C.‐B. Jeong , H.‐M. Kang , Y. H. Lee , M.‐S. Kim , J.‐S. Lee , J. S. Seo , M. Wang , J.‐S. Lee , Environ. Sci. Technol. 2018, 52, 11411. 30192528 10.1021/acs.est.8b03211

[44] J. Bhagat , N. Nishimura , Y. Shimada , J. Hazard. Mater. 2021, 405, 123913. 33127190 10.1016/j.jhazmat.2020.123913

[45] M. Mofijur , S. F. Ahmed , S. M. A. Rahman , S.k. Y. Arafat Siddiki , A. B. M. S. Islam , M. Shahabuddin , H. C. Ong , T. M. I. Mahlia , F. Djavanroodi , P. L. Show , Environ. Res. 2021, 195, 110857. 33581088 10.1016/j.envres.2021.110857

[46] M. Cole , P. Lindeque , E. Fileman , C. Halsband , R. Goodhead , J. Moger , T. S. Galloway , Environ. Sci. Technol. 2013, 47, 6646. 23692270 10.1021/es400663f

[47] Y. M. M. Paing , Y. Eom , G. B. Song , B. Kim , M. G. Choi , S. Hong , S. H. Lee , Sci. Total Environ. 2024, 924, 171681. 38490422 10.1016/j.scitotenv.2024.171681

[48] M. C. González‐Caballero , M. de Alba González , M. Torres‐Ruiz , P. Iglesias‐Hernández , V. Zapata , M. C. Terrón , M. Sachse , M. Morales , R. Martin‐Folgar , I. Liste , A. I. Cañas‐Portilla , Chemosphere 2024, 355, 141815. 38556182 10.1016/j.chemosphere.2024.141815

[49] R. Marfella , F. Prattichizzo , C. Sardu , G. Fulgenzi , N. Engl. J. Med. 2024, 390, 900. 38446676 10.1056/NEJMoa2309822 PMC11009876

[50] Y. Li , Z. Wang , B. Guan , Environ. Res. 2022, 204, 112134. 34597658 10.1016/j.envres.2021.112134

[51] M. Kosuth , S. A. Mason , E. V. Wattenberg , PLoS One 2018, 13, 0194970. 10.1371/journal.pone.0194970 PMC5895013 29641556

[52] Y. Huang , K. K.i Wong , W. Li , H. Zhao , T. Wang , S. Stanescu , S. Boult , B. van Dongen , P. Mativenga , L. Li , J. Hazard. Mater. 2022, 424, 127404. 34736178 10.1016/j.jhazmat.2021.127404

[53] Y. Liu , Y. Hu , C. Yang , C. Chen , W. Huang , Z. Dang , Water Res. 2019, 163, 114870. 31336206 10.1016/j.watres.2019.114870

[54] H. Cai , E. G. Xu , F. Du , R. Li , J. Liu , H. Shi , Chem. Eng. J. 2021, 410, 128208.

[55] Z. A. Ganie , N. Khandelwal , E. Tiwari , N. Singh , G. K. Darbha , J. Hazard. Mater. 2021, 417, 126096. 34229390 10.1016/j.jhazmat.2021.126096

[56] E. Tiwari , N. Singh , N. Khandelwal , F. A. Monikh , G. K. Darbha , J. Hazard. Mater. 2020, 397, 122769. 32422514 10.1016/j.jhazmat.2020.122769

[57] R. Amanna , M. Samavi , S. K. Rakshit , in Biotechnology and Bioengineering (Eds: G. Mannina , A. Pandey , R. Sirohi ), Elsevier, Amsterdam 2023, pp. 293–314.

[58] G. Zhou , X. Huang , H. Xu , Q. Wang , M. Wang , Y. Wang , Q. Li , Y. Zhang , Q. Ye , J. Zhang , Sci. Total Environ. 2022, 820, 153190. 35051471 10.1016/j.scitotenv.2022.153190

[59] H. Zhao , X. Huang , L.u Wang , X. Zhao , F. Yan , Y. Yang , G. Li , P. Gao , P. Ji , Chem. Eng. J. 2022, 430, 133122.

[60] N.a Zhu , Q. Yan , Y. He , X. Wang , Z. Wei , D. Liang , H. Yue , Y. Yun , G. Li , N. Sang , J. Hazard. Mater. 2022, 433, 128756. 35358818 10.1016/j.jhazmat.2022.128756

[61] A. Gabet , C. Guy , A. Fazli , H. Métivier , C. de Brauer , M. Brigante , G. Mailhot , Sep. Purif. Technol. 2023, 317, 123877.

[62] S. Kim , A. Sin , H. Nam , Y. Park , H. Lee , C. Han , Chem. Eng. J. Adv. 2022, 9, 100213.

[63] A. Fazli , M. Brigante , A. Khataee , G. Mailhot , Appl. Surf. Sci. 2021, 559, 149906.

[64] Z. Yang , Y. Li , G. Zhang , Chemosphere 2024, 357, 141939. 38621489 10.1016/j.chemosphere.2024.141939

[65] L. Dai , Z. Lei , Y. Cao , M. Zhang , X. Song , G. Wang , G. Ma , T. Zhao , J. Ren , J. Environ. Chem. Eng. 2024, 12, 112261.

[66] M. Adeel , V. Granata , G. Carapella , L. Rizzo , J. Hazard. Mater. 2024, 465, 133102. 38070270 10.1016/j.jhazmat.2023.133102

[67] K. Bule Možar , M. Miloloža , V. Martinjak , F. Radovanović‐Perić , A. Bafti , M. Ujević Bošnjak , M. Markić , T. Bolanča , M. Cvetnić , D. Kučić Grgić , Š. Ukić , Water 2024, 16, 673.

[68] Q. Chen , L. Wan , H. Zhou , F. Luo , L. Lei , N. Wang , J. Water Process Eng. 2023, 56, 104343.

[69] A. Fazli , S. Lauciello , R. Brescia , R. Carzino , A. Athanassiou , D. Fragouli , Appl. Catal. B Environ. Energy 2024, 353, 124056.

[70] G. Pulido‐Reyes , L. Magherini , C. Bianco , R. Sethi , U. von Gunten , R. Kaegi , D. M. Mitrano , J. Hazard. Mater. 2022, 436, 129011. 35643007 10.1016/j.jhazmat.2022.129011

[71] J. Nieto‐Sandoval , R. Ammar , C. Sans , Chem. Eng. J. Adv. 2024, 19, 100621.

[72] Y. Li , C. Zhang , C. Shen , G. Jiang , B. Guan , Environ. Res. 2023, 220, 115220. 36608764 10.1016/j.envres.2023.115220

[73] L. Tian , Q. Chen , W. Jiang , L. Wang , H. Xie , N. Kalogerakis , Y. Ma , R. Ji , Environ. Sci. Nano 2019, 6, 2907.

[74] Y. Cai , F. Chen , L. Yang , L. Deng , Z. Shi , Water 2023, 15, 1920,.

[75] P. García‐Muñoz , P. H. Allé , C. Bertoloni , A. Torres , M. U. de la Orden , J. M. Urreaga , M.‐A. Dziurla , F. Fresno , D. Robert , N. Keller , J. Environ. Chem. Eng. 2022, 10, 108195.

[76] C. A. Rojas‐Guerrero , M. Villanueva‐Rodríguez , J. L. Guzmán‐Mar , A. Hernández‐Ramírez , E. I. Cedillo‐González , F. E. Longoria Rodríguez , L. Hinojosa‐Reyes , J. Environ. Chem. Eng. 2023, 11, 110415.

[77] C. di Luca , J. Garcia , D. Ortiz , M. Munoz , J. Carbajo , Z. M. de Pedro , J. A. Casas , J. Environ. Chem. Eng. 2023, 11, 110755.

[78] H. Luo , Y. Zeng , Y. Zhao , Y. Xiang , Y. Li , X. Pan , J. Hazard. Mater. 2021, 413, 125342. 33618270 10.1016/j.jhazmat.2021.125342

[79] V. P. Kelkar , C. B. Rolsky , A. Pant , M. D. Green , S. Tongay , R. U. Halden , Water Res. 2019, 163, 114871. 31351353 10.1016/j.watres.2019.114871

[80] A. Khataee , A. Fazli , M. Fathinia , F. Vafaei , Ozone Sci. Eng. 2019, 41, 35.

[81] A. Khataee , A. Fazli , M. Fathinia , F. Vafaei , J. Clean. Prod. 2018, 186, 475.

[82] N. Ormeno‐Cano , J. Radjenovic , J. Hazard. Mater. 2022, 431, 128462. 35220123 10.1016/j.jhazmat.2022.128462

[83] L. Seid , D. Lakhdari , M. Berkani , O. Belgherbi , D. Chouder , Y. Vasseghian , N. Lakhdari , J. Hazard. Mater. 2022, 423, 126986. 34461534 10.1016/j.jhazmat.2021.126986

[84] M. Feilizadeh , S. H. Kochaki , M. R. K. Estahbanati , M. Kiendrebeogo , P. Drogui , Mar. Pollut. Bull. 2025, 213, 117621. 39892060 10.1016/j.marpolbul.2025.117621

[85] B. Jiang , J. Zheng , S. Qiu , M. Wu , Q. Zhang , Z. Yan , Q. Xue , Chem. Eng. J. 2014, 236, 348.

[86] J. Ren , J. Li , Y. Zhen , J. Wang , Z. Niu , Sep. Purif. Technol. 2022, 290, 120832.

[87] M. R. Winburn , M. F. Alvarado , C. L. Cheung , Nanoscale 2025, 17, 2138. 39655503 10.1039/d4nr02498b PMC11757047

[88] A. O. Adeola , B. A. Abiodun , D. O. Adenuga , P. N. Nomngongo , J. Contam. Hydrol. 2022, 248, 104019. 35533435 10.1016/j.jconhyd.2022.104019

[89] S. A. Aransiola , M. O. Victor‐Ekwebelem , B. X. Daza , P. O. Oladoye , Y. A. Alli , A. Bamisaye , A. B. Aransiola , S. O. Oni , N. R. Maddela , Chemosphere 2025, 374, 144211. 39977960 10.1016/j.chemosphere.2025.144211

[90] M. Al‐Sid‐Cheikh , S. J. Rowland , K. Stevenson , C. Rouleau , T. B. Henry , R. C. Thompson , Environ. Sci. Technol. 2018, 52, 14480. 30457844 10.1021/acs.est.8b05266

[91] W. Fu , J. Min , W. Jiang , Y. Li , W. Zhang , Sci. Total Environ. 2020, 721, 137561. 32172100 10.1016/j.scitotenv.2020.137561

[92] L. Hildebrandt , D. M. Mitrano , T. Zimmermann , D. Pröfrock , Front. Environ. Sci. 2020, 8, 89.

[93] K. B. Gavazov , I. Hagarová , R. Halko , V. Andruch , J. Mol. Liq. 2019, 281, 93.

[94] P. Wu , Y. Tang , G. Cao , J. Li , S. Wang , X. Chang , M. Dang , H. Jin , C. Zheng , Z. Cai , Anal. Chem. 2020, 92, 14346. 32880171 10.1021/acs.analchem.0c01928

[95] D. Magrì , P. Sánchez‐Moreno , G. Caputo , et al., ACS Nano 2018, 12, 7690. 29944342 10.1021/acsnano.8b01331

[96] F. Lionetto , C. E. Corcione , A. Rizzo , A. Maffezzoli , Polymers 2021, 13, 3745. 34771306 10.3390/polym13213745 PMC8587476

[97] A. Fazli , M. Brigante , A. Khataee , G. Mailhot , Chemosphere 2022, 291, 132920. 34798115 10.1016/j.chemosphere.2021.132920

[98] D. Magrì , M. Veronesi , P. Sánchez‐Moreno , V. Tolardo , T. Bandiera , P. P. Pompa , A. Athanassiou , D. Fragouli , Environ. Pollut. 271, 116262. 10.1016/j.envpol.2020.116262 33360657

[99] D. Jia , Q. Li , K. Hanna , G. Mailhot , M. Brigante , Environ. Pollut. 2021, 288, 117728. 34247005 10.1016/j.envpol.2021.117728

[100] L. D. B. Mandemaker , F. Meirer , Angew. Chem., Int. Ed. 2023, 62, 202210494. 10.1002/anie.202210494 PMC10100025 36278811

[101] X.‐D. Sun , X.‐Z. Yuan , Y. Jia , L.‐J. Feng , F.‐P. Zhu , S.‐S. Dong , J. Liu , X. Kong , H. Tian , J.‐L.u Duan , Z. Ding , S.‐G. Wang , B. Xing , Nat. Nanotechnol. 2020, 15, 755. 32572228 10.1038/s41565-020-0707-4

[102] Y.‐S. Chang , S.‐H. Chou , Y.‐J. Jhang , T.‐S. Wu , L.‐X. Lin , Y.‐L. Soo , I.‐L. Hsiao , Sci. Total Environ. 2022, 814, 152675. 34968609 10.1016/j.scitotenv.2021.152675

[103] T. Yang , J. Luo , B. Nowack , Environ. Sci. Technol. 2021, 55, 15873. 34784483 10.1021/acs.est.1c04826

[104] X. X. Han , R. S. Rodriguez , C. L. Haynes , Y. Ozaki , B. Zhao , Nat. Rev. Methods Primer 2021, 1, 87.

[105] L. Xie , K. Gong , Y. Liu , L. Zhang , Environ. Sci. Technol. 2023, 57, 25. 36576086 10.1021/acs.est.2c07416

[106] P. Li , Q. Li , Z. Hao , S. Yu , J. Liu , J. Environ. Sci. 2020, 94, 88. 10.1016/j.jes.2020.03.057 32563491

[107] D. Materic , A. Kasper‐Giebl , D. Kau , M. Anten , M. Greilinger , E. Ludewig , E. van Sebille , T. Röckmann , R. Holzinger , Environ. Sci. Technol. 2020, 54, 2353. 31951124 10.1021/acs.est.9b07540 PMC7031848

[108] J. Delgado‐Gallardo , G. L. Sullivan , P. Esteban , Z. Wang , O. Arar , Z. Li , T. M. Watson , S. Sarp , ACS EST Water 2021, 1, 748.

[109] M. Meyns , F. Dietz , C.‐S. Weinhold , H. Züge , S. Finckh , G. Gerdts , Anal. Methods 2023, 15, 606. 36644945 10.1039/d2ay01036d

[110] L. Nigamatzyanova , R. Fakhrullin , Environ. Pollut. 2021, 271, 116337. 33383415 10.1016/j.envpol.2020.116337

[111] W. Zhang , Q. Wang , H. Chen , Front. Environ. Sci. Eng. 2021, 16, 11.

